# Searching for the Rules of Electrochemical Nitrogen Fixation

**DOI:** 10.1021/acscatal.3c03951

**Published:** 2023-11-02

**Authors:** Romain Tort, Alexander Bagger, Olivia Westhead, Yasuyuki Kondo, Artem Khobnya, Anna Winiwarter, Bethan J. V. Davies, Aron Walsh, Yu Katayama, Yuki Yamada, Mary P. Ryan, Maria-Magdalena Titirici, Ifan E. L. Stephens

**Affiliations:** † Department of Chemical Engineering, Imperial College London, London SW7 2AZ, U.K.; ‡ Department of Physics, Technical University of Denmark, Kongens Lyngby 2800, Denmark; § Department of Materials, Imperial College London, London SW7 2AZ, U.K.; ∥ Osaka University, SANKEN (The Institute of Scientific and Industrial Research), Mihogaoka, Osaka, Ibaraki 567-0047, Japan

**Keywords:** nitrogen fixation, nitrogen reduction, ammonia synthesis, electrosynthesis, lithium-mediated, solid-electrolyte interphase, nitride, HSAB principle

## Abstract

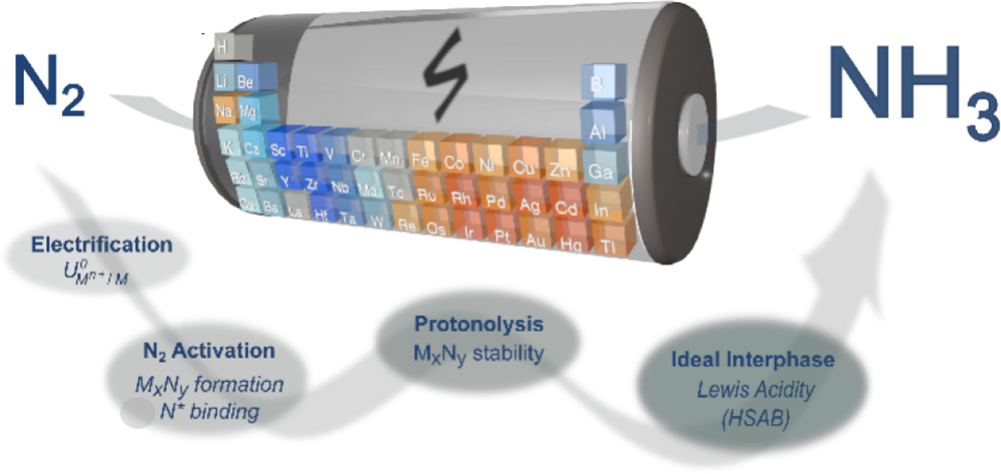

Li-mediated ammonia synthesis is, thus far, the only electrochemical method for heterogeneous decentralized ammonia production. The unique selectivity of the solid electrode provides an alternative to one of the largest heterogeneous thermal catalytic processes. However, it is burdened with intrinsic energy losses, operating at a Li plating potential. In this work, we survey the periodic table to understand the fundamental features that make Li stand out. Through density functional theory calculations and experimentation on chemistries analogous to lithium (e.g., Na, Mg, Ca), we find that lithium is unique in several ways. It combines a stable nitride that readily decomposes to ammonia with an ideal solid electrolyte interphase, balancing reagents at the reactive interface. We propose descriptors based on simulated formation and binding energies of key intermediates and further on hard and soft acids and bases (HSAB principle) to generalize such features. The survey will help the community toward electrochemical systems beyond Li for nitrogen fixation.

Thermochemical ammonia synthesis via Haber–Bosch delivers fertilizers at global-scale effectively feeding half the world.^1,2^ It is, however, a significant contributor to anthropogenic greenhouse gas emissions, accounting for ∼1% of the global emissions (500 Mt CO_2,eq_/year).^3,4^ Ambient electrochemical ammonia synthesis has potential in that regard as it could be powered by renewables in decentralized units. However, this technology is still in its nascent stages. On a solid electrode, lithium-mediated ammonia synthesis remains the only system–thus far–capable of reducing nitrogen to ammonia selectively.^5^ Since it was rigorously verified,^6^ significant breakthroughs were made,^7−9^ alongside reports uncovering the fundamental reasons behind its selectivity, likely due to the solid-electrolyte interphase, or SEI.^10−13^ This interphase is commonly associated with Li-ion batteries, where a Li-ion conducting but electrically insulating layer forms on the negative electrode from the partial breakdown of the electrolyte, stabilizing the electrolyte kinetically.^14−16^ For ammonia synthesis, it is hypothesized that a similar layer forms and selectively slows down reagents’ diffusion to the electrode, preventing substantial parasitic H_2_ evolution.^8,11,13^ In other words, a selective barrier on the electrode controls the delivery of reagents to the active surface and provides ideal species activities for NH_3_ to form. This last sentence depicts a concept that sounds transferable to alternative chemistries. Thus, why have we not found another system to reduce N_2_ on a solid electrode? This is worth asking considering that Li is identified as a critical material by the European Union Critical Materials Act^17,18^ and the inherent energy efficiency losses from the operation at Li-plating potential (−3.27 *V vs*. RHE in 0.6 M LiClO_4_ in THF and EtOH 1% v/v).^19^ In this work, we provide a systematic theory-informed method to establish trends across the periodic table and pinpoint elements that may activate N_2_. We will identify and test chemistries analogous to Li, namely, Na, Ca, Mg, compare their behavior to Li, and provide explanations for its singularity.

## 1 Theory – A Survey of the Periodic Table

**Scheme 1:**
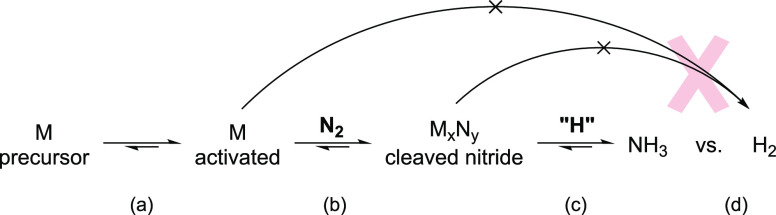
Hypothetic Reaction Pathway for Selective Nitrogen Reduction to Ammonia (a) Element electrochemical activation (e.g. reduction), (b) cleaved nitride formation (M_*x*_N_*y*_) meaning that nitrogen atoms in the structure are separated, (c) conversion to NH_3_ from a source of hydrogen (H^+^, M_*x*_H_*y*_, ...), and (d) selective over H_2_ evolution.

Let us draw a hypothetical reaction pathway that is, to date, valid for lithium and broad enough to be applied to other elements in the periodic table (Scheme 1).^20^

**Figure 1:**
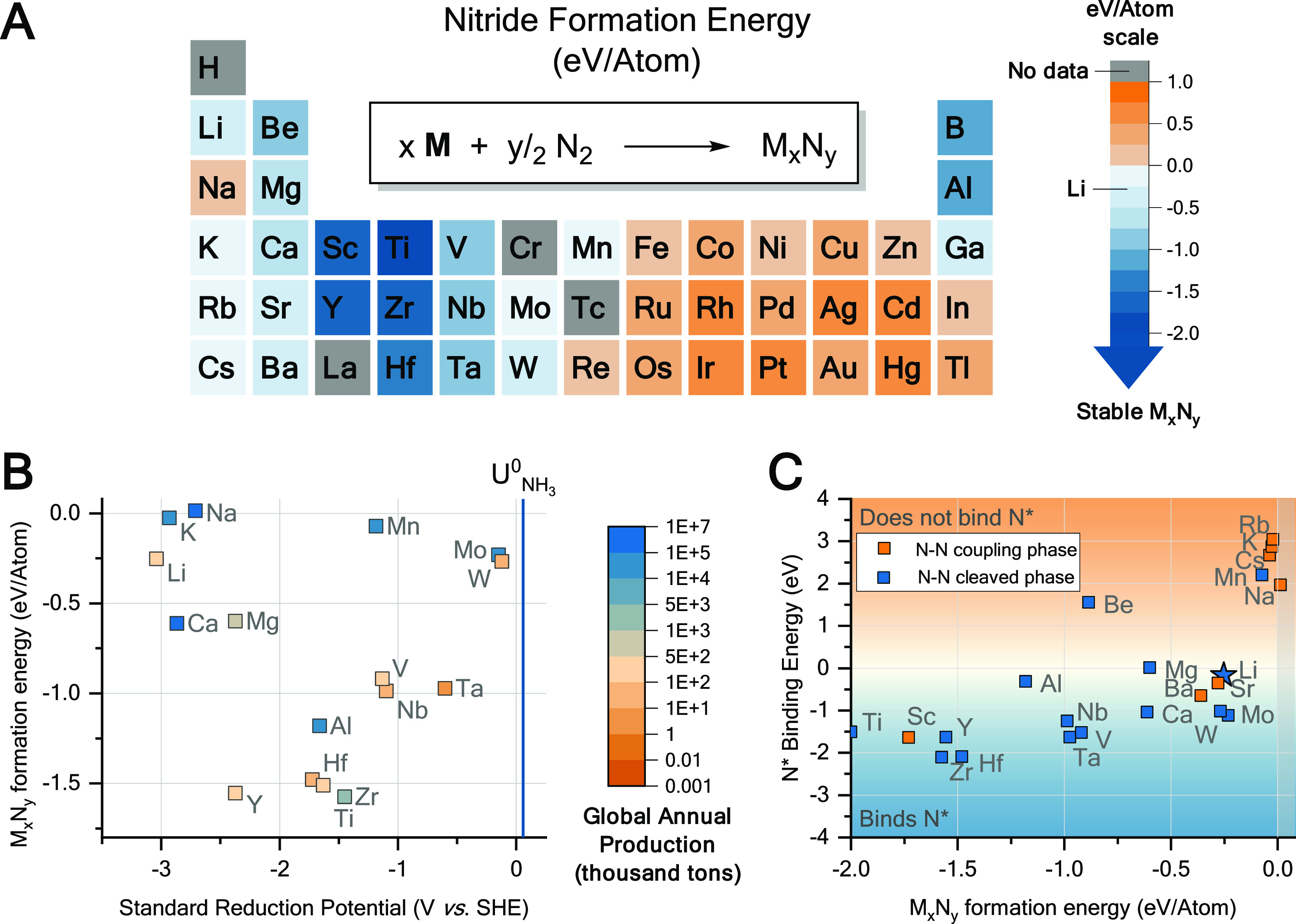
Screening chemical elements through the formation energy of metal nitrides and the standard reduction potential. (A) Periodic table of the elements (cut) and their respective nitride formation energies calculated by DFT (metal nitride stoichiometries in Table S1, La and Tc were omitted due to a missing basis set and Cr N* binding energy due to being energetically outliers). (B) Standard reduction potential of elements^24−27^ vs M_*x*_N_*y*_ formation energy, standard potential for N_2_ reduction to NH_3_ given for reference, blue line.^19^ Color scale represents the global productions of the minerals associated with these elements.^28^ (C) N* binding energy of elements calculated by DFT, plotted against their nitride formation energy.

The main hypothesis is that ammonia synthesis goes through a dissociative pathway through a cleaved nitride intermediate M_*x*_N_*y*_. In this sense, “cleaved” refers to the nitride structure having each nitrogen atom separated and surrounded with metal atoms, whereas “coupling” structures such as sodium azide (NaN_3_), is a phase which does not split N_2_. This is debatable, even for Li where intermediates are unknown,^21^ although we expect it to be a mixture of Li (M_*x*_), hydride (M_*x*_H_*y*_), nitride (M_*x*_N_*y*_), and nitride hydride (M_*x*_N_*y*_H_*z*_).^22^ However, M_*x*_N_*y*_ is likely to be a key intermediate considering its thermodynamic stability.^23^ About half of the evaluated elements have a stable nitride phase (Figure 1A, blue elements), among which Li has the most negative reduction potential,^24−27^ hence the highest intrinsic overpotential for ammonia synthesis (Figure 1B). Replacing Li with any element will likely result in high gains in energy efficiency, and some elements (e.g., Al, Ca, Mo, W, and Mg) are also produced globally at much higher rates, meaning better scalability.^28^

Nitride formation alone leaves us with many candidates. However, a material must bind nitrogen (N*, where * denotes a bond to the surface), which is linked through BEP (Bro̷nsted–Evans–Polanyi) relations^29^ to the ability for nitrogen dissociation at room temperature. This is a step required to generate a nitride (Figure 1C). Many elements can both make a nitride and bind N* (blue zone). However, some of them do not cleave N_2_ (Sc, Ba, Sr). Li is in a special spot with a close to neutral binding energy (−0.168 eV), and metal nitride formation energy (−0.25 eV/atom) (binds not too weakly nor strongly, following Sabatier’s principle), and Mg, Ca, W, and Mo come close. This screening pinpoints 12 elements, all with a nitride formation energy more negative than or equivalent to the hydride (Table S1). Ca, Mg, and Al (with Na which does not bind N*) are all in the region of negative N* binding energy and M_*x*_N_*y*_ formation energy alongside Li and have also been studied in the context of electrochemical energy storage. The flourishing field of “beyond-Li” batteries is therefore a pertinent space to explore, with working examples of nonaqueous electrolytes and, sometimes, analogous solid–electrolyte interphases.^30−32^

## 2 Experimental Electrolytic Screening in Lithium-like Electrolytes

Like in Li batteries and nitrogen reduction,^11,12,33,34^ an excessive number of parameters can influence the performance of beyond-lithium electrolytes making a holistic screening challenging. For comparative purposes, we put our theoretical screening to the test by revisiting the Li-based electrolytes with their Na, Ca, and Mg equivalents using a model electrolyte: a NTf_2_ salt dissolved in an ether solvent (tetrahydrofuran (THF) or 1,2-dimethoxyethane (DME) for Mg(NTf_2_)_2_) at different concentrations (0.1 to 1 M) and EtOH contents (0.2 to 5% v/v). While this electrolyte was used in Na,^35−37^ Mg,^31,38−40^ and Ca batteries,^31,41−43^ Al only operates on a different chemistry based on ionic liquids,^44^ hence an Al candidate being left out. DME was chosen as a solvent for Mg(NTf_2_)_2_ because the Mg salt is hardly soluble in THF, DME is a solvent of choice for Mg batteries,^31,39^ and it works for Li-mediated ammonia synthesis.^45^ Na is a control experiment to test the hypothesis that a battery-like system would not produce ammonia if it cannot bind N* and make a cleaved nitride (Figure 1C). Note that Tsuneto et al. in their seminal work compared Li-mediated ammonia synthesis against Na, with no success.^46^ On the other hand, Mg and Ca tick all the boxes so far. Comparing their reactivity with Li can either yield a new catalyst for ammonia synthesis or give us clues about its distinctiveness.

**Figure 2:**
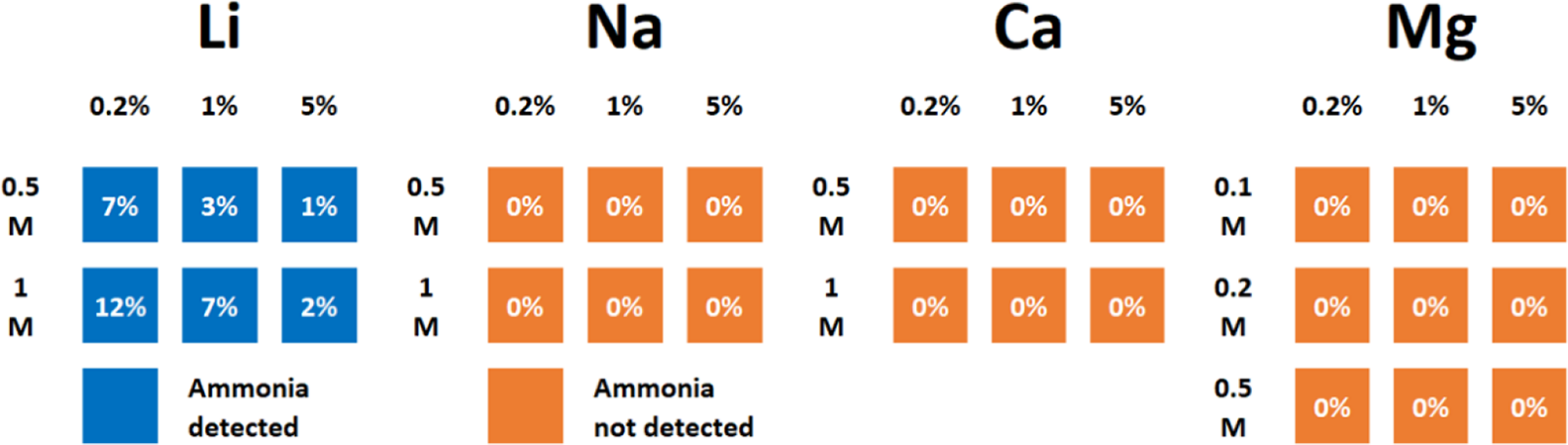
Faradaic efficiency to ammonia of different electrolytes tested electrochemically. Varying M(NTf_2_)_*n*_ salt concentrations (rows, mol·L^–1^ or M) and EtOH contents (columns, % v/v, 0.2, 1, and 5% correspond to 0.034, 0.171, and 0.857 mol·L^–1^, respectively). Solvent is DME for Mg electrolytes and THF for the rest. Electrochemical testing consists of a linear potential sweep down to a cutoff current density of −2 mA cm^–2^_geo_ followed with chronopotentiometric electrolysis at that current, passing 10 C of charge.

Selected electrolytes were tested electrochemically as described in the Supporting Information. Unfortunately, in this restricted experimental parameter space, while Li electrolytes always yield ammonia, none of the alternative metals were active (Figure 2). Why is that? It is likely that Mg and Ca chemistries are as sensitive as Li toward electrolyte modifications,^12,34^ and a restricted experimental parameter space may contribute to false-negative observations.

## 3 Why is Li so Special? – Interpretation and Model Experiments

**Figure 3:**
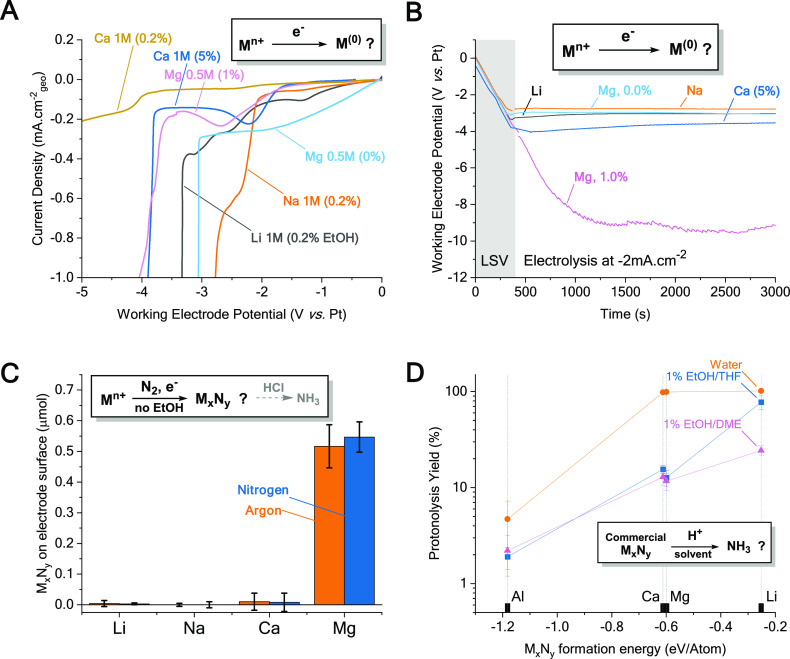
Model experiments for the identification of limiting steps in ammonia synthesis. (A) Linear sweep voltammograms (LSV) on Mo foil showing cation reduction for each element in selected electrolytes: M(NTf_2_)_*n*_ in THF (or DME for Mg electrolytes), salt concentration on plot labels with EtOH content (% v/v) between parentheses. Voltammograms for Ca electrolytes show electrode passivation when too little EtOH is present (0.2% v/v). Full electrolytic setup and conditions described in the Supporting Information (10 mV cm^–1^ sweep from open circuit to −5 V vs reference or until 1 mA cm^–2^ current density is reached, IUPAC plotting convention, *iR* corrected). (B) Recorded potentials for the same electrolytic systems during LSV and following constant current electrolysis, showing electrode passivation in Mg electrolytes containing EtOH. (C) Nitride detected following the electrolysis of electrolytes with 0.5 M salt and no EtOH saturated with argon or ^14^N_2_. Nitrides were quantified by hydrolysis of the electrode in 4 M HCl and colorimetric UV–vis quantification of the resulting solution. (D) Yield of protonolysis (log scale) of commercial nitrides (Li_3_N, Ca_3_N_2_, Mg_3_N_2_, and AlN) from their suspension in solutions containing different proton sources, calculated from the quantification of ammonia from such solution.

From Scheme 1, these catalysts should (a) plate to their metallic state, (b) make a cleaved nitride from N_2_, which (c) reacts with protons to make ammonia, and (d) selectively vs H_2_. Here, we design model experiments to determine which steps are impeding the road to success (Figure 3).

### 3.1 Are We Plating Metals?

All the tested electrolytes were subjected to a linear voltage sweep (LSV, Figure 3A) followed with constant current electrolysis (Figure 3B) to plate metals. During the LSV, the steep increase in current suggests that the metals do plate–at least in one of the tested electrolyte compositions–as expected.^37,39,42^ The overpotential to metal plating sometimes observed (e.g., Mg electrolytes) could be attributed to the use of a Mo current collector not being an ideal substrate for metal nucleation and plating.^47^ While a better substrate could be suggested for plating such metals, this was not the scope of our study and did not prevent us from plating metals. Nevertheless, plating from Ca or Mg electrolytes often generates a passivating layer that does not conduct Mg/Ca-ions, preventing continuous electrodeposition.^31,39,42,48,49^ We observe this passivation through a large increase in cell voltage in Ca and Mg systems (Figure 3A,B), for different reasons. For Ca, passivation is observed with low amounts of EtOH (up to 0.2% v/v). Better performance may be expected from Ca(BF_4_)_2_ or Ca(BH_4_)_2_ electrolytes for instance, which show Ca plating with less resistive interfaces/interphases.^41,42^ When more EtOH is introduced, this does not occur. Whether EtOH disrupts the passivating layer^13^ or takes over and evolves H_2_ is hard to tell so far. Mg however can only plate without EtOH present. Mg is highly sensitive to oxygenated protic species^31,48,50^ (e.g., water or EtOH); their contact with Mg metal yields oxides that do not conduct Mg ions. This mismatch of Ca and Mg with EtOH creates a likely hindrance in the downstream NH_3_ generation.

### 3.2 Are We Splitting N_2_?

**Figure 4:**
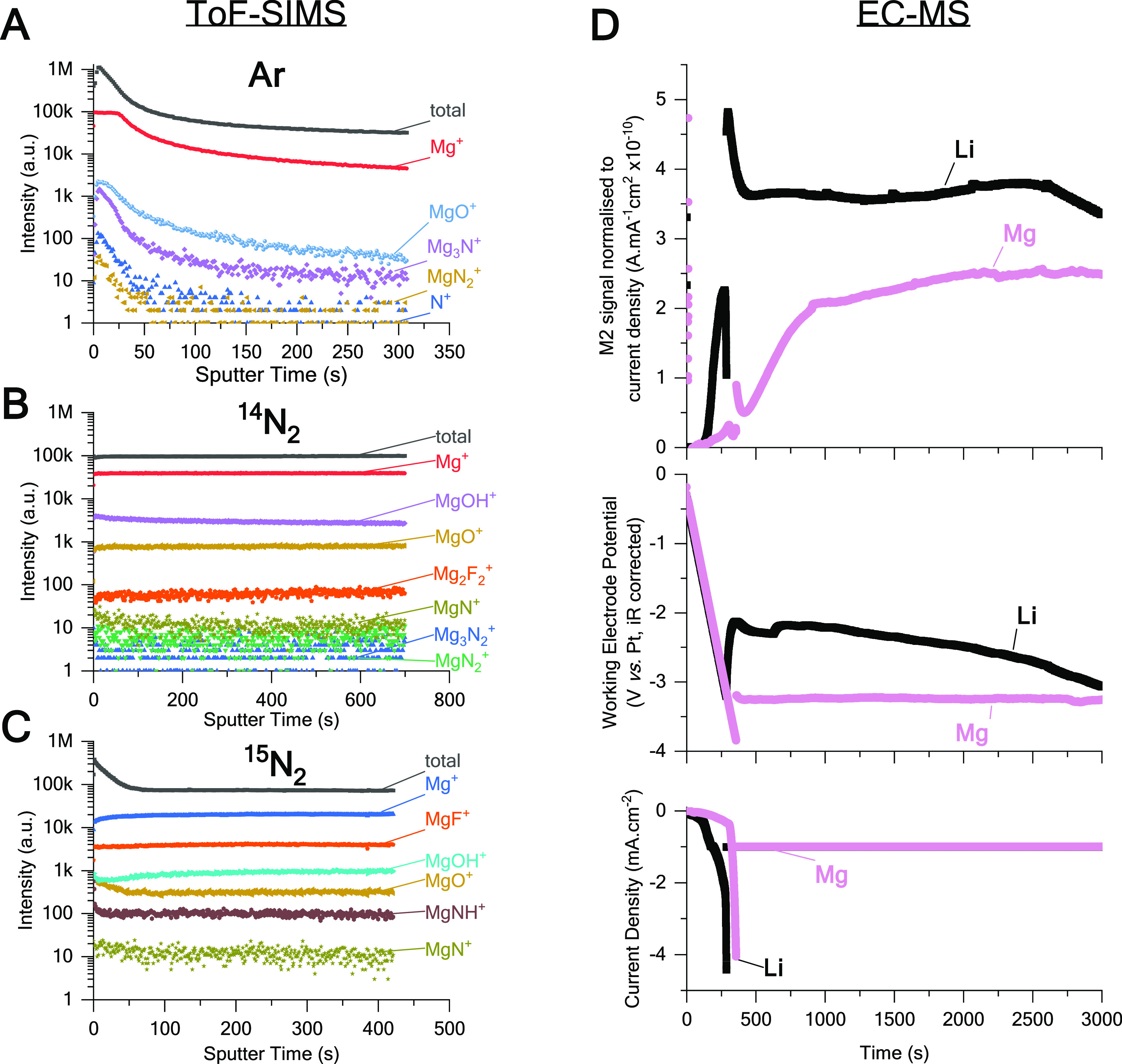
Mg electrochemistry characterization using ToF-SIMS (time-of-flight–secondary ion mass spectrometry) and EC-MS (electrochemistry–mass spectrometry). (A–C) ToF-SIMS analysis of Mg electrodes post electrolysis in (A) Ar, (B) ^14^N_2_, and (C) ^15^N_2_, displaying Mg_*x*_N_*y*_ fragments detected from positive ion sputtering, whether or not N_2_ gas was present during electrolysis (details in Figure S6). (D) EC-MS in-line detection of generated H_2_ during electrolysis of a LiNTf_2_ or Mg(NTf_2_)_2_ 0.5 M in DME (conditions in Figure S7), showing a larger signal for *m*/*z* = 2 (likely H_2_, normalized to current density) during linear sweep voltammetry and initial stages of constant current electrolysis (top plot), but also a largely negative potential for the Mg medium electrolysis (middle plot).

Cleaved nitride formation from N_2_ is energetically favorable on Li, Ca, and Mg. Extensive literature describes nitrides synthesis in specific conditions.^2,51−54^ To verify it in our system, we performed identical electrolysis experiments in an aprotic medium, to favor nitride formation over proton reduction, if it were to happen. *Post-mortem* hydrolysis of the electrode deposits and ammonia quantification suggest that a nitride can be formed only on a Mg surface (Figure 3C). This is corroborated with ToF-SIMS (time-of-flight–secondary ion mass spectrometry) analysis of an electrode *post-mortem* (Figure 4 and Figure S6), showing traces of Mg_*x*_N_*y*_ fragments (*x* = 1,2,3 and *y* = 1,2). However, equivalent amounts of nitride are generated in the absence of N_2_ (Figure 3C), and similar Mg_*x*_N_*y*_ fragments observed by ToF-SIMS, suggesting that they come from the breakdown of NTf_2_^–^ ions (setup proved for other N-contaminants). ^15^N_2_ isotope labeled experiments confirm the nitride’s adventitious nature (Figure S5). This is a valuable reminder of the countless false positives in nitrogen fixation.^6,55,56^ To understand the inactivity of Mg toward N_2_, we performed the same electrolysis in an *operando* electrochemistry–mass spectrometry setup that allows subsecond detection of volatile species,^57,58^ and compare gas evolution from an electrolyte made of Mg(NTf_2_)_2_ or LiNTf_2_ 0.5 M in DME. The *m*/*z* = 2 (M2) signal, most likely corresponding to H_2_, was plotted against time of electrolysis, intentionally left as is (as opposed to being converted to H_2_ flux) to emphasize the raw and semiquantitative aspect of such information. This signal is less intense in the Mg system vs Li (Figure 4D, 6–7 times lower initially (*t* = 400–500 s)). This suggests that the Mg surface is not only less reactive to N_2_ but also to sources of H. This difference becomes less pronounced as the time of electrolysis passes, and we believe that it is due to the lower volume and shorter interelectrode distance in this setup (vs the standard electrochemical cell used to screen electrolytes). As time goes on, H^+^ generated at the counter electrode may build up and alter electrolyte composition,^58^ hence the initial working conditions being more representative of the processes undergoing in the original cell. Our ToF-SIMS analysis of the Mg electrode (Figure 4 and Figure S6), displays traces of MgF and MgO species, which are reported to passivate Mg metal in batteries.^31,48,59^ The extreme potentials at which the working electrode goes during electrolysis (>1 V over Mg reduction potential) goes with this idea, alike the Li system in unoptimized conditions.^11^ Presumably, this passivation renders the electrode more inert than Li.

### 3.3 Are We Generating Ammonia?

Nitrides conversion to ammonia was completed in specific conditions (high temperatures, plasmas, etc.).^23,54,60,61^ We verify whether this limits ammonia synthesis in our conditions by trying to protonolyze commercial nitrides in different protic media (Figure 3D). The protonolysis yield scales with the nitrides formation energy and with their Gibbs free energies of hydrolysis calculated by Gao et al.^23^ This suggests that a nitride that is easy to make will be harder to take all the way to ammonia. When repeating this experiment in water, proton activity is drastically increased; Ca and Mg nitrides fully hydrolyze, although still not enough to fully convert AlN – the most stable of them, to NH_3_. This might be one reason why Li works better than others considering its near-neutral nitride formation energy (Figure 1A). We acknowledge however that transport of reactants through the SEI likely adds a layer of complexity to this analysis.^13,34^

### 3.4 Are We Doing It Selectively?

According to our electrolytic screening, the short answer is no. Mg and Ca can, in principle, make stable nitrides (Figure 1A), which readily decompose to ammonia (Figure 3D). However, nothing other than Li was able to generate ammonia. The research community opinion converges on the idea that control over the SEI structure and composition is crucial for selective ammonia synthesis.^8,11,13,62^ What is so unique about the Li SEI then? Why cannot analogous elements form an active SEI? While substantial progress has been made in chemistries beyond Li, it is not a simple substitution.^30,36^ The Li battery SEI is unique in its stability as opposed to the more soluble Na SEI,^63^ and in its ability to conduct active ions (vs Mg and Ca).^31,64^ In batteries, the focus is on maximizing Li^+^ transport through the SEI while preventing electrons to reach the electrolyte,^14^ but matters may be different in the context of ammonia synthesis. We expect the active surface to be buried under the SEI, consistent with recent work.^13,65,66^ The electron-insulating role of the SEI remains essential for selective ammonia synthesis, but it should moderate the transport of Li^+^ and H^+^ and maximize the access of N_2_ and NH_3_.^67^ As such, the ideal SEI composition is likely different from the one a battery needs; hence, the translation of Li–and beyond Li–chemistries are not straightforward.

## 4 The Ideal Interphase

The investigations herein highlight that to make ammonia, we need an SEI (or similar) that can regulate the transport of key reactants to the active surface. However, we do not have a way to compare it across the periodic table because it is so specific to lithium. Or do we? It is known that the composition of the SEI is a direct consequence of the environment of metal ions, namely their (de)solvation,^68−70^ which also applies to Li-mediated ammonia synthesis.^8,11^ One can rationalize the interaction of metal cations with their environment as Lewis acid/base controlled, where the metal cation is an acid that binds a base (solvent, anion). The Hard and Soft Acid and Bases (HSAB) principle defines and classifies chemical species’ acidity/basicity based on their atomic structure to describe their interaction and reactivity.^71^ In metal-air batteries, solvent stability and effects on O_2_ reduction were rationalized through HSAB,^72,73^ and changes in battery performance were attributed to a change in metal cations acidity.^74^ In Li metal batteries, the incorporation of K^+^ ions altered the SEI composition because of their softer character.^75^ It sounds reasonable to use the HSAB principle as a descriptor for interfaces and interphases. How do we scale it? Klopman quantified the acidity of acids through quantum mechanics,^76^ returning a successful descriptor defined by the difference between the LUMO energy of said acid and their energy of desolvation from water (eq 1):
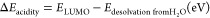
1

**Figure 5:**
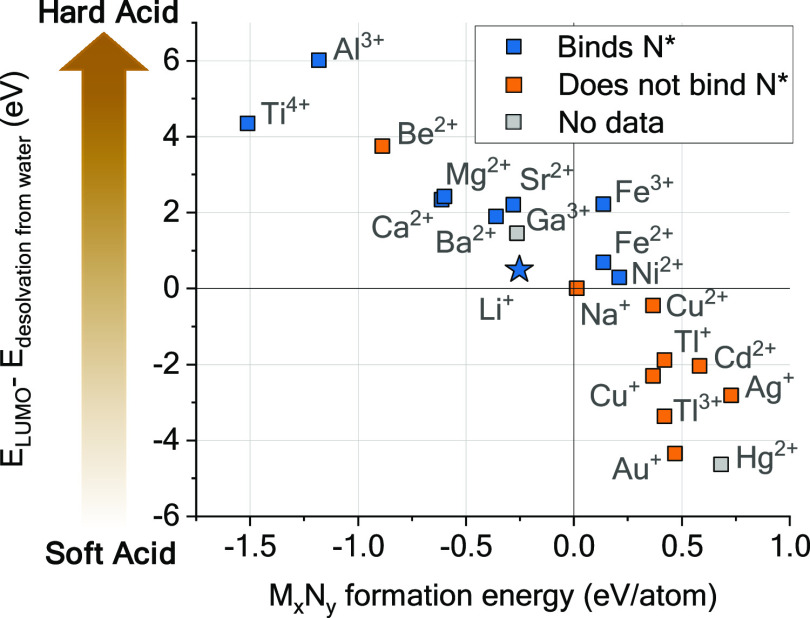
Using Hard and Soft Acids and Bases principle as a descriptor for solid electrolyte interphase and nitrogen fixation. Plotting Klopman’s descriptor for acid hardness–softness^76^ against the formation energy of metal nitrides.

This is especially relevant since these are the two main processes at the origin of metal reduction and SEI formation.^69,77,78^ We plotted the cation hardness against the nitride formation energy of their respective elements to test their relation (Figure 5).

This correlation suggests that the nitride formation energy relates to the HSAB principle, although we cannot assign the underlying causation. We observe that Li has the lowest acidity among the elements capable of making a cleaved nitride. This analysis is not absolute, and we note that the acidity scale depicted in Figure 5 is calculated in water, a hard base. However, changing cations environment to a softer one will affect their acidity, making the scale dynamic.^79^ A working example is the equilibrium electrochemical potential of metal with metal ions, affected by the environment of such ions.^78,80^ Take a LiN(SO_2_F)_2_ 1 M electrolyte, switch solvent from THF to diglyme (softer base), and the Li equilibrium potential goes more negative by 90 mV, a result of a change in Li^+^ acidity and interfacial reactivity.^78^ This way, elements such as Ca, Mg, or Al could move on this acidity scale when their environment is altered and may potentially be just as successful as Li at making ammonia. Notably, a few elements (e.g., Mo and W) are not presented in Figure 5, since they were absent from the current analysis work.^76^ These elements fulfill the energetic criteria in Figure 1C and are reported as promising candidates alongside their nitrides. Several groups have used Mo electrodes to reduce N_2_ to NH_3_:^5,6,11,12,46,62,65,67^ however, they only reduce N_2_ at potentials sufficiently negative for Li to plate (i.e., the Mo merely serves as a current collector).^19,80^ There could be several reasons for the apparent inactivity of Mo. First, the ability to form a nitride is a necessary but insufficient criterion: an SEI that balances reagents at the active surface is also needed.^67^ Second, all studies to date have used Mo that would have been previously exposed to air and hence covered with a native oxide,^81^ the surface of which would have a reactivity to N_2_ distinct from metallic Mo. We encourage further studies to explore such elements (Ca, Mg, Al, Mo, W, etc.) and discover the appropriate interphase that will generate ammonia selectively on these electrodes.

**Scheme 2:**
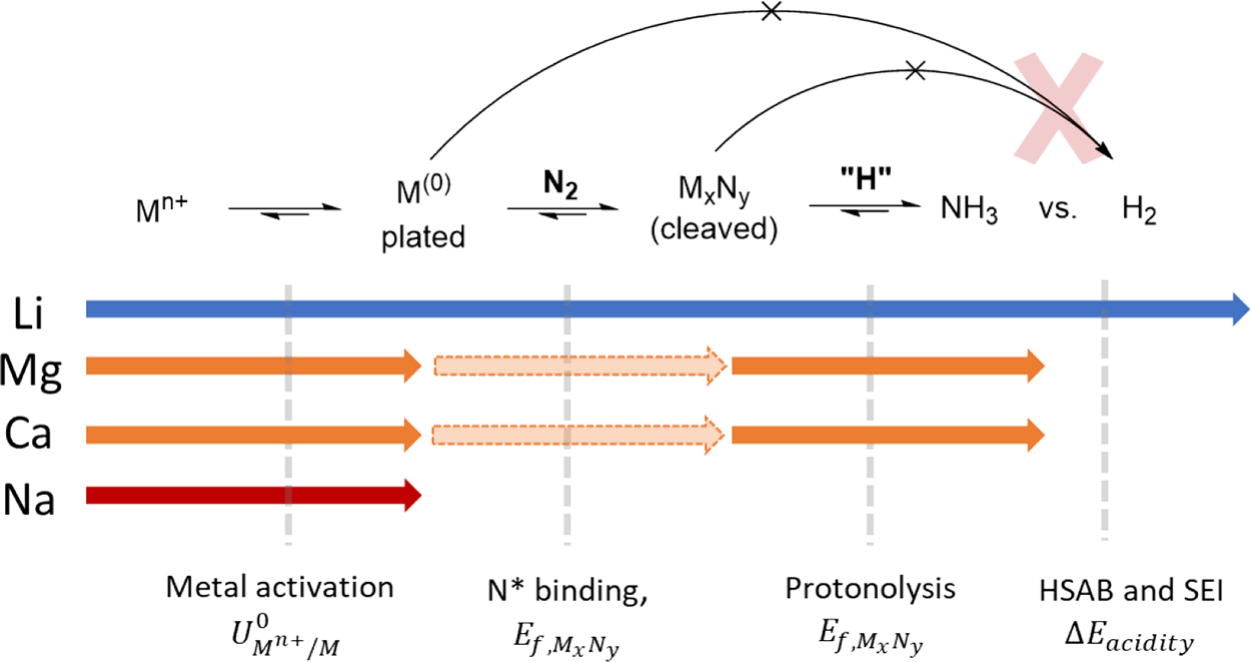
Bottlenecks in Ammonia Synthesis on Lithium-like Heterogeneous Electrochemical Systems and Suggested Descriptors to Understand and Solve N_2_ Activation Beyond Li

In summary, we have demonstrated a systematic method to explore catalytic systems for the electrochemical fixation of N_2_, to address and understand the distinctiveness of Li as the only solid–electrode mediator for electrochemical ammonia synthesis. Through a combination of theory and experiments, we tested elements analogous to Li (Na, Ca, and Mg; Scheme 2).

We propose that Li is unique in several ways: (i) it binds N* not too weakly/strongly; (ii) it makes a cleaved nitride phase which (iii) is not too stable and readily protonolyzes to NH_3_; (iv) its solid–electrolyte interphase is optimal for ammonia synthesis since it conducts Li ions, moderates access to protons, and protects the active surface from H* poisoning to yield ammonia. Just like nitride formation or N* binding, we suggest this last feature to be generalizable across the periodic table using the HSAB principle, where Lewis acidity guides toward optimal interfaces and interphases. The failure of Mg and Ca to produce ammonia in the conditions tested here does not preclude them from forming the appropriate basis for ammonia synthesis in the appropriate environment. We hope this analysis can inspire the community to design viable alternatives to the Li-mediated ammonia synthesis.

## Supporting Information Available

Materials; experimental methods (electrochemistry, quantification, isotope labeled studies setup, results of the electrolytic screening of Li, Na, Ca, and Mg electrolytes, ToF-SIMS characterization of Mg electrodes, electrochemistry–mass spectrometry study of Mg and Li electrolytes) and respective accompanying figures; tabled results of the DFT calculations, with literature data on standard reduction potentials, minerals global production and HSAB acidity scale; DFT calculation scripts (https://github.com/AlexanderBagger/Beyond_Li_N2_reduction, GitHub repository) (PDF)

## ABBREVIATIONS

SEI: solid–electrolyte interphase
M_*x*_N_*y*_: metal nitride
M_*x*_H_*y*_: metal hydride
BEP: Bro̷nsted–Evans–Polanyi
NTf_2_: bis(trifluoromethanesulfonyl)imide
THF: tetrahydrofuran
DME: 1,2-dimethoxyethane
LSV: linear sweep voltammogram
LUMO: lowest unoccupied molecular orbital
HSAB: Hard and Soft Acids and Bases
