# Correction to “Searching for the Rules of Electrochemical Nitrogen Fixation”

**DOI:** 10.1021/acscatal.4c00448

**Published:** 2024-02-14

**Authors:** Romain Tort, Alexander Bagger, Olivia Westhead, Yasuyuki Kondo, Artem Khobnya, Anna Winiwarter, Bethan J. V. Davies, Aron Walsh, Yu Katayama, Yuki Yamada, Mary P. Ryan, Maria-Magdalena Titirici, Ifan E. L. Stephens

Page 14514

Two different plane wave cutoffs were used in the DFT calculations. This is standard practice (see refs 1–3 in the original paper), as it provides an optimal balance of speed and accuracy for both types of simulations: 800 eV for bulk simulations and 500 eV for adsorption energies. Mistakenly, we used the same N_2,(g)_ reference for both simulations, whereas we should have used separate references for each (the equivalent of this mistake in an electrochemical experiment is an incorrect calibration of a reference electrode). Correcting the N_2,(g)_ reference with 500 eV yields a constant offset of 0.526 eV in stronger binding for all *N adsorption energies. In addition, the nitride formation energy reported for titanium was incorrectly reported to be at −2.0 eV/atom while the calculated value is of −1.51 eV/atom. Finally, upon revisiting the calculations, we were able to converge the *N binding energy of Cr, which did not converge until then because of spin polarization effects.

These three corrections were updated in Figure 1, which should now read as given here.

**Figure 1:**
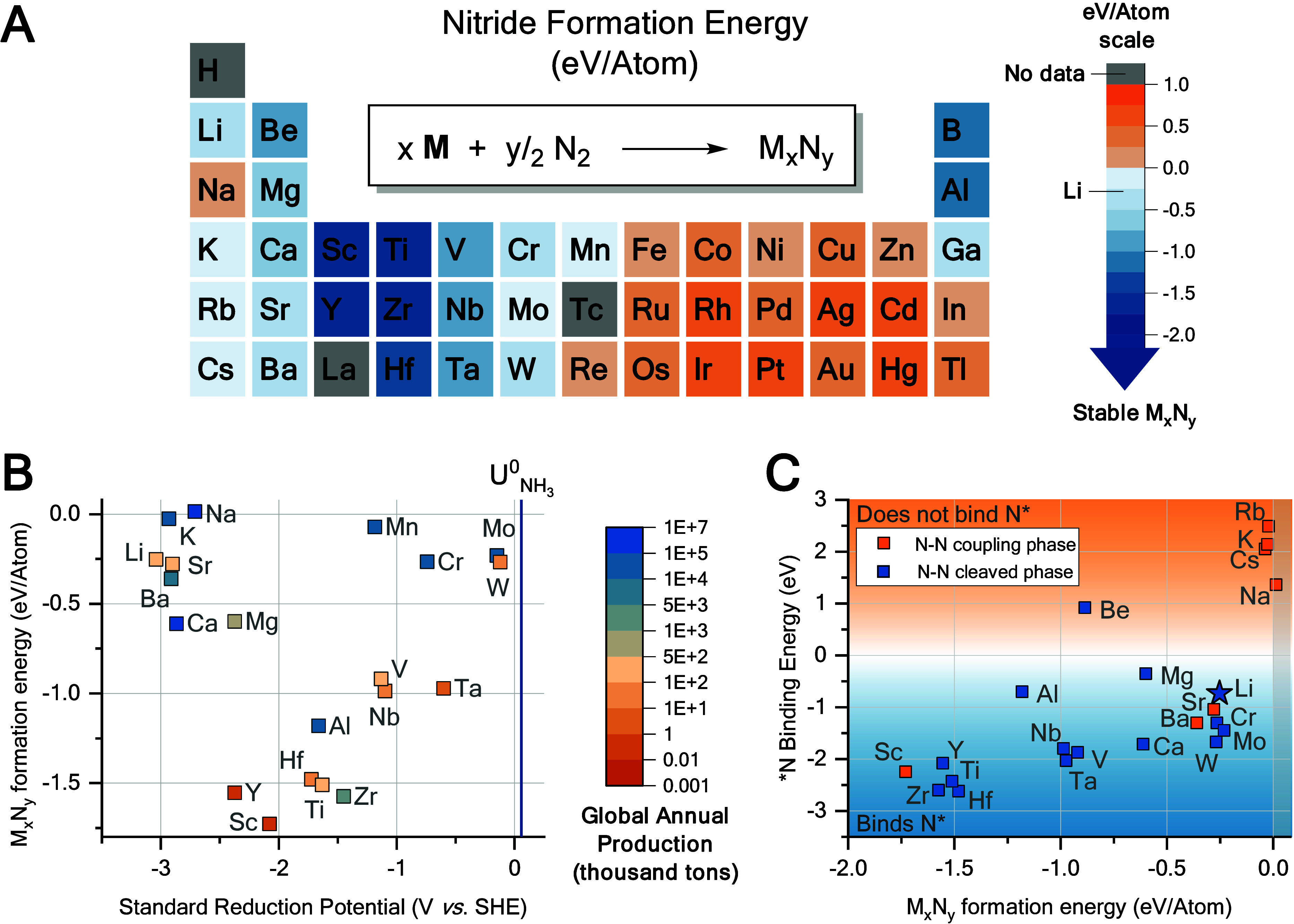
Screening chemical elements through the formation energy of metal nitrides and the standard reduction potential. (A) Periodic table of the elements (cut) and their respective nitride formation energies calculated by DFT. Metal nitride stoichiometries in Table S1, La and Tc were omitted due to missing basis set. (B) Standard reduction potential of elements^24–27^ vs M_*x*_N_*y*_ formation energy, standard potential for N_2_ reduction to NH_3_ given for reference, blue line.^19^ Color scale represents the global productions of the minerals associated with these elements.^28^ (C) *N binding energy of elements calculated by DFT, plotted against their nitride formation energy.”

Following the DFT correction, the main text was updated to account for the change in Li *N binding energy and include Cr in the analysis. In the paragraph above section 2:

“Li is in a special spot with a close to neutral binding energy (−0.734 eV) and metal nitride formation energy (−0.25 eV/atom) (Li binds not too weakly nor strongly, following Sabatier’s principle), and Mg, Ca, W, Cr, and Mo come close. This screening pinpoints 13 elements, all with a nitride formation energy more negative or equivalent to the hydride (Table S1).”

Page 14518, paragraph after Figure 5. Two sentences were changed to include Cr in the analysis:

**Figure 5:**
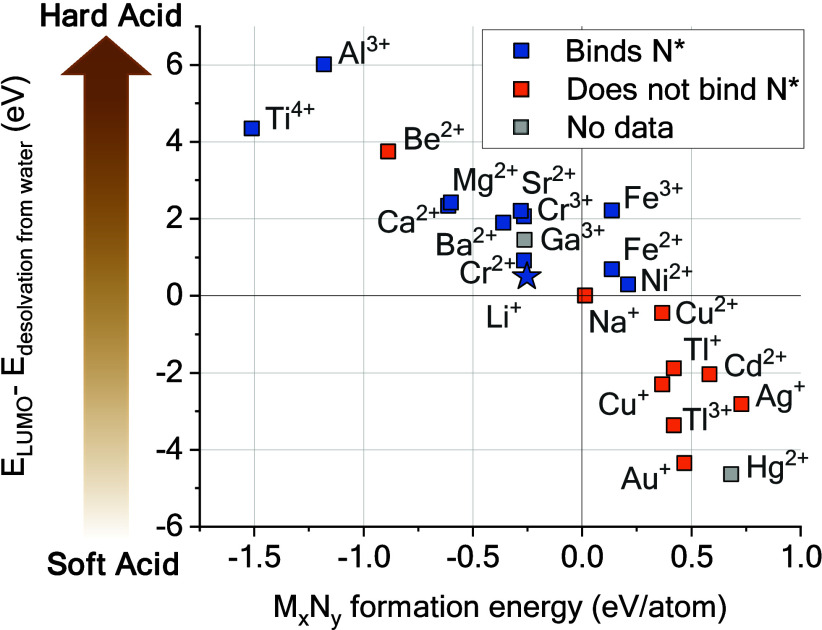
Using Hard and Soft Acids and Bases principle as a descriptor for solid electrolyte interphase and nitrogen fixation. Plotting Klopman’s descriptor for acid hardness–softness^76^ against the formation energy of metal nitrides.

“Notably, a few elements such as Cr (interestingly close to Li in Figure 5) or Mo and W which are not presented in Figure 5, since they were absent from the current analysis work,^76^ are close to Li in energetic properties.”

“We encourage further studies to explore such elements (Ca, Mg, Al, Mo, W, Cr, ...) and discover the appropriate interphase that will generate ammonia selectively on these electrodes.”

Data points for Cr were added to Figure 5, which now reads as given here.

The errors associated with DFT calculations were corrected in the Supporting Information:

•Page 21: “2x2x4” replaced with “2x2x5”

•Page 32–33: Table S1 updated with correct simulation values.

Finally, our simulation data have been updated on GitHub accordingly (https://github.com/AlexanderBagger/Beyond_Li_N2_reduction).

