# Supplementary material for: Searching for the Rules of Electrochemical Nitrogen Fixation
Source: ACS Catal. 2023 Nov 2;13(22):14513–22. doi: 10.1021/acscatal.3c03951 (PMC10660346; doi:10.1021/acscatal.3c03951)
Supplement: Supplementary file 1 — cs3c03951_si_001.pdf [file cs3c03951_si_001.pdf]

Supplementary Information

Searching for the Rules of Electrochemical Nitrogen Fixation

Romain Tort[1], Alexander Bagger[1,2†], Olivia Westhead[3], Yasuyuki Kondo[4], Artem Khobnya[3], Anna Winiwarter[3], Bethan J. V. Davies[3], Aron Walsh[3], Yu Katayama[4], Yuki Yamada[4], Mary P. Ryan[3], Maria-Magdalena Titirici[1], Ifan E. L. Stephens[3*]*

1 Department of Chemical Engineering, Imperial College London, SW7 2AZ London, UK

2 Department of Physics, Technical University of Denmark, Kongens Lyngby 2800, Denmark

3 Department of Materials, Imperial College London, SW7 2AZ London, UK

4 Osaka University, SANKEN (The Institute of Scientific and Industrial Research), Mihogaoka, Ibaraki 567-0047, Osaka, Japan

Corresponding Authors:

*Ifan E. L. Stephens – i.stephens@imperial.ac.uk – Royal School of Mines, Imperial College London, South Kensington Campus, London SW7, United Kingdom

*[†]Alexander Bagger – alexbag@dtu.dk – Present Address: Department of Physics, Technical University of Denmark, Kongens Lyngby 2800, Denmark

Table of contents

1. Materials 3

2. Methods 4

2.1. Electrolytes preparation 4

2.2. Electrolytic screening of Li, Na, Ca and Mg electrolytes 4

2.3. Ohmic drop correction 6

2.4. Isotopically labelled $^{15}N_2$ experiments 6

2.5. Sample preparation for NH_3 quantification 8

2.6. Ammonia colorimetric quantification (UV-vis) 8

2.7. Ammonia quantification by NMR (^{1}H NMR) 10

2.8. Nitride formation model experiments (**Figure 3C**) 11

2.9. Commercial nitride protonolysis model experiments (**Figure 3D**) 12

2.10. ToF-SIMS analysis of the Mg electrodes surface (**Figure 4**) 12

2.11. Electrochemistry-Mass Spectrometry experiments (**Figure 4D**) 13

2.12. Density functional theory simulations 14

3. Figures 15

Figure S 1. Electrochemical cell used in the screening of electrolytes for ammonia synthesis. 15

Figure S 2. Ohmic drop determination and correction method for electrochemical potential measurements. 16

Figure S 3. Gas recycling setup with electrochemical cell for isotopically labelled nitrogen reduction experiments, using $^{15}N_2$, $^{14}N_2$ or Ar. 17

Figure S 4. Ammonia quantification via salicylate standard addition – data analysis. 19

Figure S 5. ^{1}H NMR quantification of $^{14}NH_3$ and $^{15}NH_3$. 20

Figure S 6. Time-of-Flight - Secondary Ion Mass Spectrometry (ToF-SIMS) depth profiling of the electrode deposits obtained after electrolysis of $Mg(NTf_2)_2$ 0.5M in DME. 21

Figure S 7. Schematic of the Electrochemistry – Mass Spectrometry cell for in line gas evolution analysis. 23

4. Tables 24

Table S 1. Summary of the theoretical data gathered from the literature or calculated. 24

1. Materials

Mo foil (≥99.9 %, 125 µm thick), Cu foils (≥99.9 %, 0.5 mm thick), Pt foil (≥99.95 %, 25 µm thick) and Pt mesh (≥99.9 %, 60 µm thick) were purchased from Goodfellow Cambridge. Bis(trifluoromethanesulfonyl)imide (NTf_2) salts were purchased from Solvionic Inc. (Li: ≥99.9%, < 20ppm water ; Ca: ≥99.5%, < 250ppm water ; Mg: ≥99.5%, < 250ppm water, Na: ≥99.5%, < 20ppm water). Ethanol (≥99.5 %, Extra Dry, absolute, AcroSeal™), Potassium Nitrite (≥97%, Thermo Scientific Chemicals) were bought from Thermofischer™. Tetrahydrofuran (THF) (anhydrous, ≥99.9 %, inhibitor-free), 1,2-Dimethoxyethane (DME) (anhydrous, ≥99.5%, inhibitor-free), HCl (30 %, Suprapur®), H_2SO_4 (96%, Suprapur®), HCl (solution 4.0M in 1,4-dioxane), (Ammonium Chloride (ReagentPlus®, ≥99.5%), Ammonium-^{15}N chloride (≥98 atom % ^{15}N, ≥99%), Lithium Nitride (−60 mesh, ≥99.5%), Calcium Nitride (powder, -200 mesh, 99% trace metals basis), Magnesium Nitride (cubic phase, −325 mesh, ≥99.5% trace metals basis), Aluminium Nitride (powder, -200 mesh, 99.8% trace metals basis), Sodium Salicylate ($C_6H_4(COONa)(OH)$, ReagentPlus®, ≥99.5%), D_2O (99.9% D, MagniSolv™ for NMR spectroscopy), Methanol (suitable for HPLC, ≥99.9%), Potassium Nitrate (ACS Reagent, ≥99.0%), Potassium Hydroxide Solution (45 wt. % in H_2O) and Nitrite Test kit (photometric,

0.002-1.00 mg/L (NO2-N), 0.007-3.28 mg/L (NO2-), Spectroquant®) were purchased from Merck KGaA Darmstadt (Sigma Aldrich). Sodium Pentacyanonitrosylferrate(III) dihydrate (ACS, 99 – 102 %) was purchased from Alfa Aesar, Sodium Hypochlorite (14% Cl_2 in aqueous solution, GPR RECTAPUR®), and Sodium Hydroxide (pellets, AnalaR NORMAPUR) were purchased from VWR. Single compartment glass cell and gas traps were custom made by Artistic and Scientific Glassware, Oxford. Single compartment PEEK cell was made in house at the Chemical Engineering workshop, using Swagelok 1/8 in. tube fittings to connect gas lines. Purifiers for the Ar and N_2 gas lines providing purity levels of H_2O, H_2, CO_2, O_2, CO, nonmethane hydrocarbon (NMHC), CH_4, NH_3, NO_x to < 0.5 ppb were purchased from NuPure. N6 Ar and N6 N_2 gas was purchased from BOC. Electrochemistry and electrolyte preparation was carried out in an Ar atmosphere glovebox (MBraun, H2O < 0.3 ppm, O2 < 0.3 ppm).

2. Methods

2.1. Electrolytes preparation

Lithium and Sodium salts were used as purchased, Calcium and Magnesium were dried under vacuum at 100°C for at least 3 days before use (Contain <50ppm water after this). Appropriate

amounts of salts were weighed in an Ar glovebox, the dissolved in a few ml of THF or DME. Appropriate amounts of EtOH were added, and the solutions was transferred to a volumetric flask, completed with solvent to reach the desired volume. Water content of such electrolyte was monitored before use by measuring the electrolyte density and sampling 1.5ml of this electrolyte to run 3 Karl Fisher measurements of the water levels, then density was used to convert ppm levels to concentration. All electrolytes made reported water content below 50ppm.

2.2. Electrolytic screening of Li, Na, Ca and Mg electrolytes

The faradaic efficiencies and voltammetry measurements reported in **Figure 2** and **Figure 3A** and **B** of the manuscript are the result of the following experiment. A 3-electrode, one compartment, sandwich cell made of PEEK was assembled as described in **Figure S1** and consists of: (i) a 1 cm^2 Mo foil working electrode, polished with 400, 1500 and 2500 grit silicon carbide paper (and wetting with ethanol) to a mirror finish, dipped in 4 M HCl and sonicated in ethanol for 10 min, mounted on a Cu current collector, (ii) a Pt mesh counter electrode on a 1 cm^2 Pt foil, (iii) a Pt mesh reference electrode mounted on a Cu contact. While $LiFePO_4$ was reported to be a more appropriate (and true) reference electrode for this system[1,2], cross-contamination would be highly detrimental to this study. To minimize its likelihood, we would

have had to prepare a new reference electrode for each experiment. This was considered a waste of material and time, taking into account the potential at which the electrolytic tests were performed was not of crucial interest so to justify the use of $LiFePO_4$. Within an Ar-filled glovebox, the cell was assembled, with the reference midway through the two other electrodes (1.8 cm between each). Gas-tight compartments were filled with 3.5 mL electrolyte (no stirring device included). A glass pre-bubbler containing THF or DME was mounted ahead of the cell to saturate the inlet gas with solvent vapour (**Figure S1**). An ammonia trap, made of HCl 0.5 M in 1,4-dioxane/THF, was set up downstream from the cell to trap any ammonia made and evaporated from the electrolyte. The setup was leak tested by passing Argon through at a rate of $10 ml.min^{-1}$. The cell was then saturated with THF-pre-saturated N6 N_2, bubbling for 45 min at a flow rate of 10 $ml.min^{-1}$, then turned down to 2 $ml.min^{-1}$. The following electrochemical procedure was performed in the Argon glovebox at room temperature, using a Biologic SP-150 potentiostat. After assessing and fitting electrolyte resistance using impedance spectroscopy (PEIS at open circuit, 20 mV oscillation, 100 kHz to 1 Hz frequency range, see fitting method in 2.3.), the working electrode potential was swept from open circuit voltage down to 5 V *vs.* Pt with a cut-off current density of -1 $mA.cm^{-2}$. After that, it was set at -2 $mA.cm^{-2}$, passing a total

of 10 C charge (1 h 23 min 20 s). Electrolyte resistance change was assessed post electrolysis using the same method as before, and the setup was purged with N6 Argon for 30min at a flow rate of 10ml.min^{-1}. Voltammograms and amperograms were corrected for ohmic drop after the experiment was finished. After experiments, the cell was rinsed with EtOH out of the glovebox and boiled in ultrapure water (>18.2 MΩ, Sartorius), then dried in a vacuum oven at 70°C. The electrolyte and the downstream trap were both sampled for ammonia quantification on the same day.

2.3. Ohmic drop correction

The ohmic drop for the working and counter electrode was assessed by electrochemical impedance spectroscopy (PEIS at open circuit, 20 mV oscillation, 100 kHz to 1 Hz frequency range), as described in previous works (**Figure S2**)[1]. Despite being the most appropriate way for us to obtain electrolyte resistance, we acknowledge that in some instances, ohmic drop might be overestimated and therefore overcompensated in some measurements, such as in **Figures 3A, B**.

2.4. Isotopically labelled $^{15}N_2$ experiments

Gas recycling setup

A gas recycling setup was constructed to perform electrolysis of a fixed volume (closed loop) of $^{15}N_2$, based on previously published works from Nielander *et. al*[3], pictured in **Figure S3**. The setup consists of a glass one-way dual action gas flow tube connected to a set of ¼” stainless steel gas lines, which are also connected to an electrochemical cell. In the glass part, two 316 stainless steel springs circle a magnetic piston (a magnet embedded in a PEEK cylinder), which drives gas flow by back-and-forth movement. The piston’s movement power and frequency are controlled using a set of copper wire coils wrapped around a plastic support that fits around the glass tube. The coils’ magnetic fields were alternated by sequentially inverting the electrical current going through them, moving the piston back and forth by induction. The gas lines have two working positions. The first “open” one lets an inlet gas go through the glass tube, the gas lines, and the electrochemical cell down to an exhaust to saturate the gas phase with a desired gas. The second “closed” position lets the gas present in the glass part and the electrochemical cell circulate through a closed loop of gas lines, to saturate the cell’s electrolyte with present gas. One can switch between working positions using a four-way valve. Switching between Ar, $^{14}N_2$ and $^{15}N_2$ can be done using a set of two-way and three-way valves ahead of the gas recycling setup. Ar and $^{14}N_2$ feeds are the same as described in previous paragraphs. $^{15}N_2$ feed (364584-

5L-EU, 98 atom % 15N) cylinder that is sequentially connected to a gas regulator, a gas purifier (identical to the one used for $^{14}N_2$ and Ar), and the recycling setup.

Cell setup and gas flow procedure for electrolysis

A single compartment glass cell with a working volume of 15 ml was used for electrolysis since the previously used sandwich cell yielded a pressure drop that was too high for us to flow gas using the recycling setup. The cell, described in previous works[4] is assembled by fitting a three electrode setup consisting of a 1cm^2 Mo working electrode mounted on a Cu wire, a 1 cm$^2_{geo}$ Pt mesh counter electrode and a Pt wire pseudo reference in between, placed such a way that the working and counter electrodes are approximately 1 cm apart, with the reference midway through. The cell was connected to the gas recycling setup, in its "open" position. N6 grade Ar gas (purified as described earlier) was passed through the setup for 20 min at a rate of 20 ml.min^{-1} to remove impurities from the glovebox atmosphere. Then, the gas inlet was switched to either $^{14}N_2$ or $^{15}N_2$, flowing at a rate of 10 ml.min^{-1} (approximate rate for $^{15}N_2$ since no mass flow controller was mounted on this inlet) for 15min to replace Ar. After that, the recycling setup was switched to its "closed" position, and the magnetic piston was activated to flow gas for 30 min. Electrolysis was then performed as described in **2.2**. Finally, the setup was switched back to its

open position, flowing Ar through the setup for 20 min at a rate of 20 $ml.min^{-1}$ and the cell was disassembled.

2.5. Sample preparation for NH_3 quantification

Non-aqueous samples (for electrolyte colorimetric NH_3 quantification): Electrolyte or gas trap post electrolysis were collected, with its total volume measured. 6 x 800 µl were collected alongside 400 µl of previously saved pristine electrolyte blank in separate vials. 40 µl of 4 M aqueous HCl was added to each one of the four vials to trap NH_3 as NH_4Cl, which were then placed in a 70˚C water bath (90˚C for DME samples) for 1 h to evaporate solvents. Each concentrate was then redissolved in 2 ml ultrapure water for colorimetric quantification (**2.6.**).

Aqueous samples (for colorimetric and NMR quantification of nitrides on electrodes): After electrolysis, the working electrode was collected and transferred to a septum vial which was taken out of the glovebox. 325 µl of aqueous HCl 4 M was added using a needled syringe to dissolve the deposited materials, then 6175 µl of ultrapure water was added to obtain a 6.5 ml sample. 400 µl of this solution was sampled, to which were added 100 µl of a solution containing 1 mM MeOH, 5% v/v HCl and 10% v/v D_2O, to be used for ^1H NMR quantification (final

solution: 200 μM MeOH, 5% v/v HCl, 2% v/v D_2O) (**2.7**). The remaining 6.1 ml were neutralized with 400 μl NaOH 4M (prepared from NaOH Suprapur®) to 6.5ml, which were used for UV-vis salicylate quantification (**2.6.**).

2.6. Ammonia colorimetric quantification (UV-vis)

Salicylate reagents preparation: A sodium hypochlorite alkaline solution was prepared by mixing sodium hypochlorite 14 % w/w and sodium hydroxide 0.4 M in a 1:9 v/v ratio. This solution must be done shortly before every quantification experiment as the stock solution stability is limited in time. A “salicylate – catalyst” solution was prepared by dissolving 40 g sodium salicylate powder in 50 ml ultrapure water, to which 1 ml of a 50 mM aqueous sodium nitroprusside ($Na_2[Fe(CN)_5NO]\cdot 2H_2O$) solution was added. Volume was completed to 100 ml in a volumetric flask, to yield a solution containing 2.5 M sodium salicylate and 0.5 mM sodium nitroprusside catalyst. The solution was stored at 5˚C in the dark for several months. Sometimes, the sodium salicylate powder was contaminated with ammonia salts impurities. To purify it from these interferents, 40 g sodium salicylate was dissolved in 300 ml DI water, to which 50 ml of 6 M aqueous HCl was added dropwise under constant stirring. The salicylic acid precipitate was filtered and washed with three times 200 ml ultrapure water, then dried under vacuum at 40˚C.

For 20 g of obtained salicylic acid, the solid was dissolved in 35 ml sodium hydroxide 4 M, to which were added 580 ml of sodium nitroprusside 50 mM solution, then completed with ultrapure water to 58 ml.

Salicylate method and standard addition: we follow a colorimetric detection method based on the complexation of ammonia with sodium salicylate to create a blue dye, described in previous works[1]. For this method, a 1 ml sample was diluted to a volume of 2 ml with ultrapure water. Then, 280 µl of the "salicylate – catalyst" solution was added, followed with 280 µl of the sodium hypochlorite alkaline solution. Samples were left to age in the dark for 45 min, then characterised by UV-vis absorption spectroscopy, measuring absorbance of light between 500 nm and 900 nm wavelengths, and measuring the difference in absorbance between the maximum (650 nm) and baseline (900 nm).

In this work, we couple this quantification experiment to the quantification method of standard addition (**Figure S4**)[1], spiking sequential amounts of a NH_4Cl standard solution (500 ppm, 9.35 mM) to the as prepared 1 ml samples and performing the salicylate quantification method to each one of these samples. By doing a linear regression on the obtained measurements, one can trace back to the ammonia present in the electrolyte, corresponding to negative of the intercept

between the linear plot and the x axis (or the ratio between the slope m of the linear regression and its intercept b) (see equations $(1-3)$). $[NH_3]$ is the ammonia concentration in the electrolyte, V_{ele} is the electrolyte volume, F is the Faraday constant, C is the total charge passed during the experiment, FE is the Faradaic Efficiency to ammonia, A is the working electrode geometric surface area.

$$(1)\ [NH_3] = \frac{b}{m} \qquad (2)\ FE = \frac{3[NH_3]V_{ele}F}{C} \qquad (3)\ Yield\ Rate = \frac{[NH_3]V_{ele}}{A.Exp\ time}$$

Variances (σ_k^2) and standard errors (s_k) in measurements are estimated using the below statistical equations. A_i is the absorbance of sample with concentration c_i, $\bar{A}$ and $\bar{c}$ are the mean absorbance and concentration, respectively. m and b are the slope and intercept of the fitted linear regression. N is the overall number of measurements.

$$\sigma_A^2 = \sum_i (A_i - \bar{A})^2 - m^2 \sum_i (c_i - \bar{c})^2 \qquad \sigma_m^2 = \frac{\sigma_A^2}{\sum_i (c_i - \bar{c})^2} \qquad \sigma_b^2 = \sigma_A^2 \frac{\sum c_i{}^2}{\sum_i (c_i - \bar{c})^2}$$

$$s_k = \sqrt{\frac{\sigma_k^2}{N-1}}\ (k = A, m, b) \qquad s_{c,NH3} = \sqrt{\frac{\sigma_{c,NH3}^2}{N-1}} = C_{NH_3}\sqrt{\left(\frac{s_b}{b}\right)^2 + \left(\frac{s_m}{m}\right)^2}$$

2.7. Ammonia quantification by NMR (^1H NMR)

Calibration curve standards preparation

Standard solutions were made to build a calibration curve. Solutions were prepared by dissolving known amounts of $^{14}NH_4Cl$ or $^{15}NH_4Cl$ in water, to which were added enough D_2O, MeOH and aqueous HCl to obtain a range of ammonium chloride solutions, with 200 mM MeOH, 5% v/v HCl and 2% v/v D_2O.

NMR procedure for ammonia quantification

Each calibration solution was then subjected to the following NMR experiment for ammonia detection. The experiments were measured on a Bruker Avance III HD 500MHz spectrometer running TopSpin3.6.5 and equipped with a z-gradient Prodigy/5mm tuneable probe and a BSMS GAB 10amp gradient amplifier providing a maximum gradient output of 53.5G/cm (5.35G/cmA). Water suppression was achieved using a standard Bruker pulse program zggpw5 which employs a Watergate W5 sequence. The gradient ratios for this double echo experiment were 34:22. The spectra were collected at a frequency of 500.13MHz with a spectral width of 9014Hz (centred on the water signal at ~4.7ppm) and 65536 data points giving an acquisition time of 3.64s. A relaxation delay of 1s was employed along with a binomial water suppression

delay of 111µs. Smoothed-square shaped (SMSQ10.100) gradient pulses were 1ms in length with a recovery delay of 200µs. Number of scans was 512 with 4 dummy scans. The data was processed with one order of zero-filling and using an exponential function with a line broadening of 0.3Hz. Obtained data and corresponding calibration curves are available in **Figure S5**.

2.8. Nitride formation model experiments (**Figure 3C**)

Electrolytes were prepared by dissolving the appropriate salt in its solvent like described above (**2.1.**), although no proton source was added. They were then subjected to the same electrochemical procedure, in the same conditions as for the electrolyte screening described above here (**2.2.**). Then, the working electrode deposit post electrolysis was hydrolysed and resulting ammonia was quantified as described (**2.5. and 2.6.**), using the salicylate colorimetric UV-vis detection method.

2.9. Commercial nitride protonolysis model experiments (**Figure 3D**)

Appropriate amounts of commercial nitrides were suspended in either EtOH 1% v/v in THF or DME (in the glovebox), or simply water (out of the glovebox). They were then left to stir at room temperature over 3 days. After this time, suspensions were left to decant for at least 5h, and

the supernatant was sampled for ammonia quantification as described (**2.5. and 2.6.**), using the salicylate colorimetric UV-vis detection method.

2.10. ToF-SIMS analysis of the Mg electrodes surface (**Figure 4**)

After electrolysis, the electrochemical cell was dismantled into the glovebox, and the working Mo electrode was put in a vial, heat sealed in moisture barrier bags (RS Components, United Kingdom) and transported to a different Ar-glovebox (H_2O < 0.1 ppm, O_2 < 0.1 ppm) where they were mounted on a back-mount sample holder and loaded into an inert atmosphere transfer suitcase. The samples were then transferred to the ToF-SIMS machine (TOF.SIMS5 IONTOF GmbH, Münster, Germany) in an Ar atmosphere. The transfer suitcase was only opened when the pressure of the loadlock chamber was lower than $3x10^{-5}$ mbar. The analysis was performed with a 25keV Bi^+ primary ion beam with the current of 1.2 pA and the high current bunched mode was applied to achieve high mass resolution. Samples were sputtered using GCIB (Gas Cluster Ion Beam) Ar_n^+(n between 1100 and 1200) at 10 nA, which is very gentle and minimises sample damage[5]. Analysis area was 200 μm x 200 μm. Sputter area was 500 μm x 500 μm (**Figure 4** and **Figure S6**). Some samples had a very uneven topography, which made it difficult to analyse the data due to difference in time-of-flight of the same fragments from different parts

of the analysis area. To mitigate this problem, smaller regions of interest were analysed which were all at the same height.

2.11. Electrochemistry-Mass Spectrometry experiments (**Figure 4D**)

All Electrochemistry-Mass Spectrometry experiments are carried out using the Spectro Inlets Electrochemistry-Mass Spectrometry System which is housed inside an N5 Argon glovebox. Electrochemistry-Mass Spectrometry allows subsecond, real-time detection of gases and volatile species in electrochemical processes[6,7]. This is possible due to a unique membrane chip that can support liquid on its topside and a gas sampling volume below which is connected to a mass spectrometer through a capillary. The membrane allows fast equilibration between the liquid and gas sampling volume. Furthermore, gases can be introduced into the sampling volume and quickly saturate the liquid. All experiments were carried out introducing N6 Nitrogen through the sampling volume of the membrane chip. The membrane and capillary are fabricated using semiconductor fabrication techniques and are contained within a thumb-sized silicon chip. The electrochemical cell sits on top of the silicon chip and has a disc-shaped working volume in which the electrodes and separators are housed. The working volume has a diameter of 9 mm and a thickness of 100 μm (**Figure S7**).

The layers inside the cell are assembled in ascending order: 8 mm diameter 25 µm Celgard® 2500 separator, 5 mm diameter 40 µm thick copper mesh (EL-Cell GmbH) 6 mm diameter 260 µm Whatman® glass microfiber filter GF/A grade. The counter electrode is a 5 mm diameter 25 µm thick Pt foil from MaTecK. A 5mm diameter stainless steel plunger is used to contact the counter electrode and apply pressure to the cell for good electrical contact. The cell has 3 x 0.8 mm diameter ports, a 0.5 mm Pt wire (acting as the reference electrode) is inserted into one and a 0.5 mm copper wire is inserted into the other, the latter contacting the copper mesh working electrode. Once assembled, the $Mg(NTf_2)_2$ or $LiNTf_2$ 0.5 M in DME electrolyte is injected into the cell using a 3 mL syringe connected to one of the ports using a Luer adapter.

Further to this, the cell was left to equilibrate at open circuit for 20min before electrochemical testing (**Figure 4D**). Electrolyte resistance was assessed by impedance spectroscopy (PEIS at open circuit, 20 mV oscillation, 100 kHz to 1 Hz frequency range, see **Figure S2** for method). The working electrode potential was swept from open circuit voltage down to a potential with a cut-off current density of -2 $mA.cm^{-2}$. Once this current was reached, it was held at this level, passing a total of 7 C charge (3500 s).

2.12. Density functional theory simulations

We carry out two sets of simulations for nitride formation energies and *N binding energies with accessible databases and analysis scripts on https://github.com/AlexanderBagger/Beyond_Li_N2_reduction.

For the Nitride formation energy and hydride formation energy we download the most stable structure and metal structure from the materials project[8]. We initiate these structures with spin and optimize them to a force below 0.05 eV/Å using the RPBE functional[9], a 800 eV planewave cutoff and an automated k-point density set to 25/cell sizes using ASE[10] and GPAW[11,12]. As each nitride and hydride structure has different stoichiometry, we calculate the formation energies as:

$$Nitride\ formation\ energy = E_{M_xN_y - M_x} * E_{M - N_y} * \frac{1}{2}E_{N_2}$$

$$Hydride\ formation\ energy = E_{M_xH_y - M_x} * E_{M - H_y} * \frac{1}{2}E_{H_2}$$

Where $E_{M_xN_y}$, $E_{M_xH_y}$ and E_M is the simulated DFT bulk energies and E_{N_2} and E_{H_2} are the molecular energy. For the N* binding energy we optimize bulk hpc, fcc and bcc structures and following build 2x2x4 structures with the three lower layers fixed. The system is then relaxed with a 500 cutoff plane wave basis set and the RPBE functional using ASE/GPAW. The binding energies are calculated as: $N^*\ binding\ energy = E_{N^*} - E_* - \frac{1}{2}E_{N_2}$

Where E_{N^*} is the relaxed slab structure with N adsorbed, E_* is the bare slab and E_{N_2} is the nitrogen molecule.

3. Figures

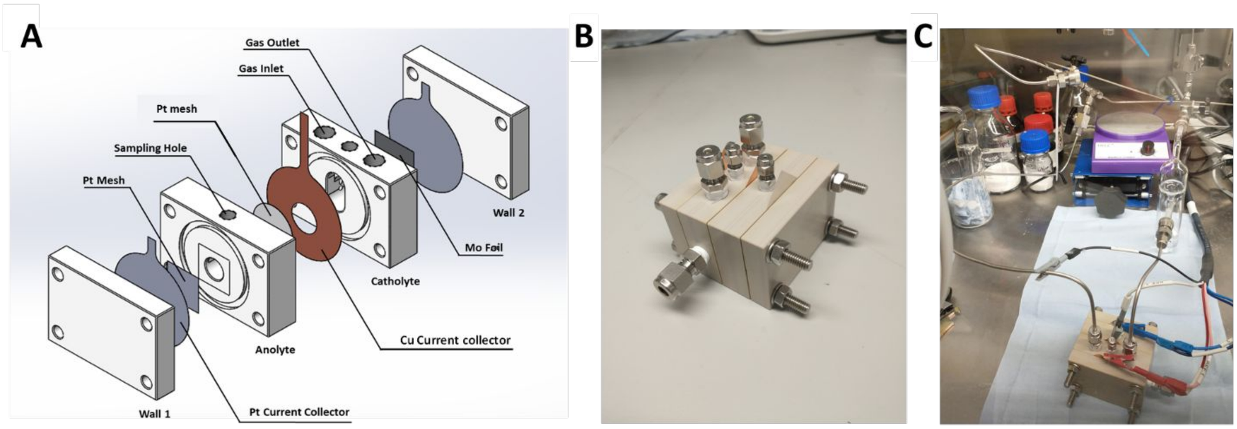

Figure S 1. Electrochemical cell used in the screening of electrolytes for ammonia synthesis.

(**A**) Cell assembly diagram: 4mL electrolyte capacity, $1cm^2$ working and counter electrodes surface areas, 1.7cm distance between each electrode, gas-tight with gas flow at ambient pressure (**B**) Assembled cell picture, (**C**) Cell in operation in a glovebox, closed gas line equipped with a THF or DME pre-bubbler to saturate inlet gas with solvent, and a gas trap made of HCl 0.5M in 1,4-dioxane/THF (made from commercial HCl 4M in 1,4-dioxane).

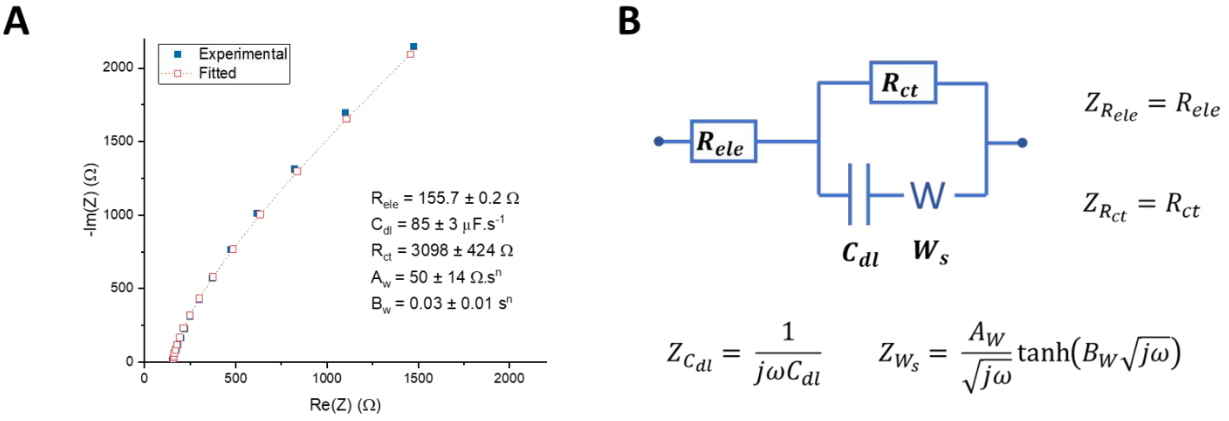

Figure S 2. Ohmic drop determination and correction method for electrochemical potential measurements.

(**A**) Example of impedance spectrum of the working electrode for the cell assembly made for electrolysis screening experiments. Recorded by potentiodynamic electrochemical impedance spectroscopy (PEIS) at open circuit voltage, with an oscillation amplitude of 20 mV at frequencies ranging from 200 kHz to 500 mHz. The ohmic drop correction was performed manually after the experiments by correcting potentials with the formula U_{eff}=$U_{recorded} - R_{ele}i$ where U_{eff} is the corrected electrode potential, $U_{recorded}$ is the raw potential measured during experiments and i is the current passed during experiments. $Rele$ is the uncompensated resistance between the working and reference electrode, collected from the PEIS measurements. R_{ele} is obtained here through fitting of the experimental data to a suitable equivalent circuit. (**B**)

Equivalent circuit used to fit the impedance spectra and extract R_{ele} as well as other impedance contributions such as: R_{ct} the charge transfer resistance, C_{dl} the double-layer capacitance, and Z_{Ws} the Warbug impedance describing diffusion processes.

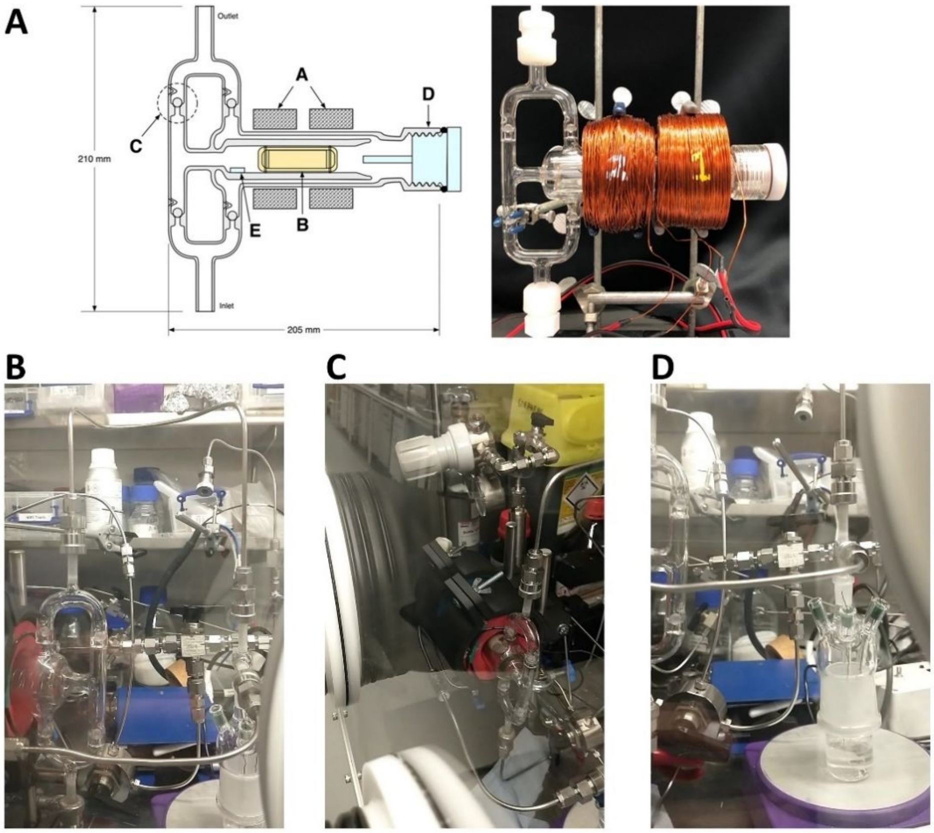

Figure S 3. Gas recycling setup with electrochemical cell for isotopically labelled nitrogen reduction experiments, using $^{15}N_2$, $^{14}N_2$ or Ar.

(**A**) Picture and sketch of the original recycling pump design. Reprinted in part with permission from Nielander, A. C.; Blair, S. J.; McEnaney, J. M.; Schwalbe, J. A.; Adams, T.; Taheri, S.;

Wang, L.; Yang, S.; Cargnello, M.; Jaramillo, T. F. Readily Constructed Glass Piston Pump for Gas Recirculation. *ACS Omega* **2020**, *5* (27), 16455–16459. https://doi.org/10.1021/acsomega.0c00742. Copyright 2020 *ACS Omega*.[3] (**B**, **C**, **D**) Pictures of the setup in the glovebox. The setup comprises a glass recycling pump (**C**) activated by an oscillating magnetic field and a PEEK piston with stainless steel springs. It comprises two gas circuits, switched to one or the other by a 4-way valve. The first "open" one flows Ar, $^{15}N_2$ or $^{14}N_2$ (interchangeably via a 3-way valve) through the pump and the electrochemical cell (**D**), purging the whole system and going to the exhaust. The second "closed" circuit recirculates the gas trapped in the pump and in the cell via the activation of the pump, to saturate the electrolyte with said gas.

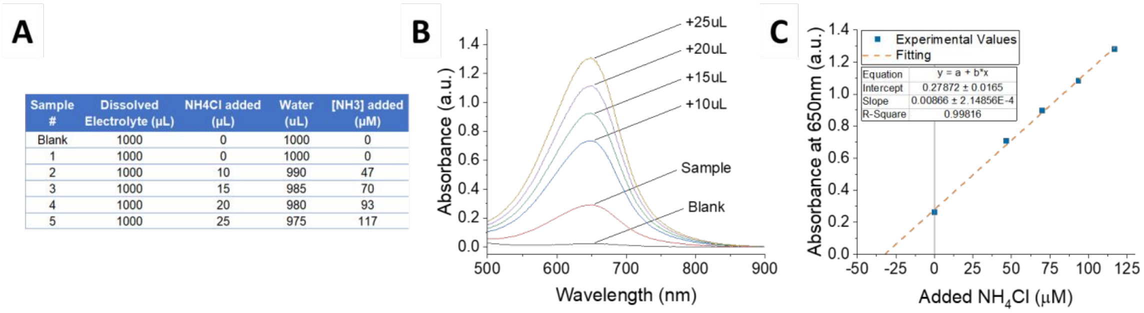

Sample #	Dissolved Electrolyte (μL)	NH4Cl added (μL)	Water (uL)	[NH3] added (μM)
Blank	1000	0	1000	0
1	1000	0	1000	0
2	1000	10	990	47
3	1000	15	985	70
4	1000	20	980	93
5	1000	25	975	117

Figure S 4. Ammonia quantification via salicylate standard addition – data analysis.

(**A**) samples preparation volumes, (**B**) resulting UV-vis spectra, (**C**) reported maxima vs. added NH_4Cl. Reprinted with permission from Tort, R.; Westhead, O.; Spry, M.; Davies, B. J. V.; Ryan, M. P.; Titirici, M.-M.; Stephens, I. E. L. Nonaqueous Li-Mediated Nitrogen Reduction: Taking Control of Potentials. *ACS Energy Lett.* **2023**, *8*, 1003–1009. https://doi.org/10.1021/acsenergylett.2c02697. Copyright **2023** *ACS Energy Letters.*[1]

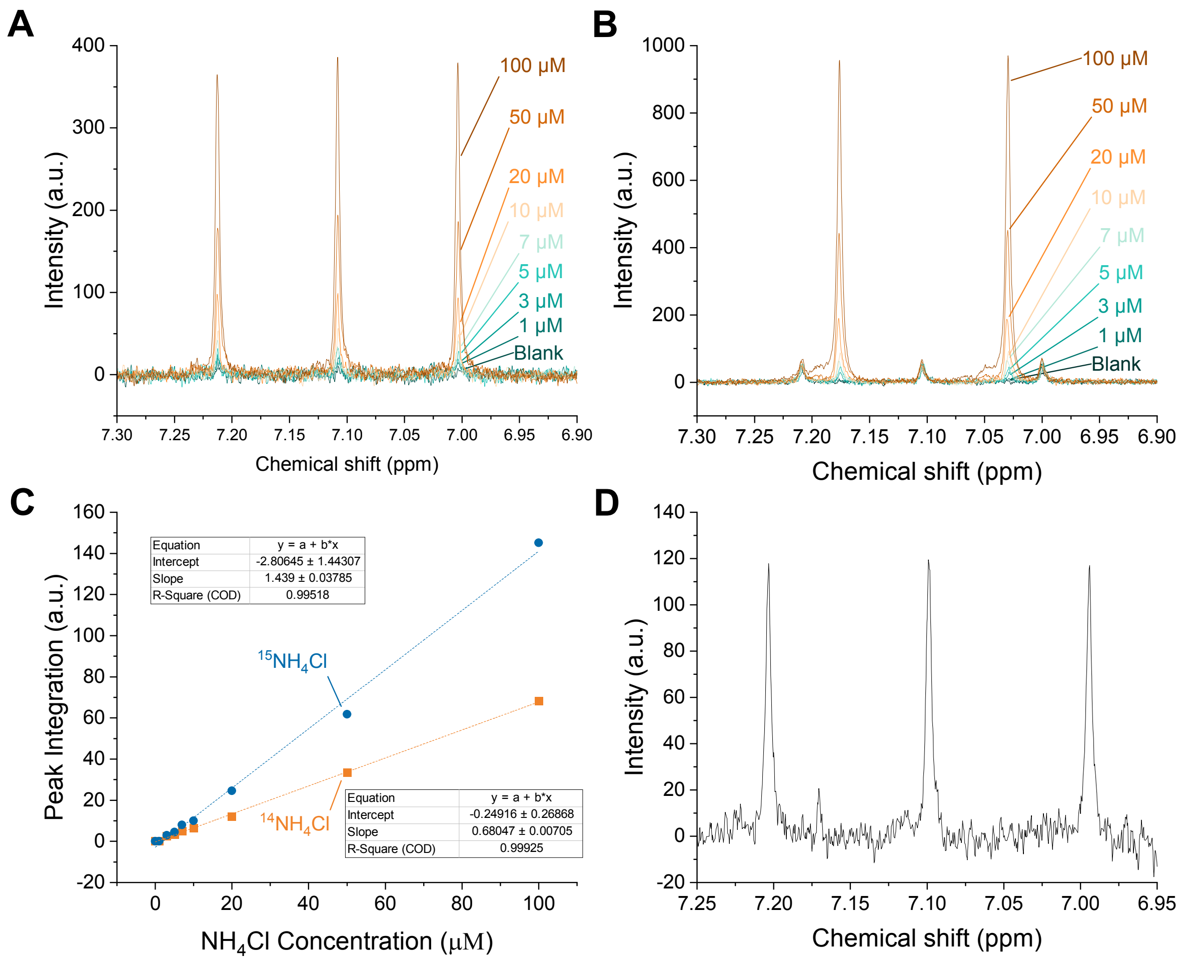

Figure S 5. ^{1}H NMR quantification of $^{14}NH_3$ and $^{15}NH_3$.

NMR signals obtained from the preparation of samples with known concentrations of (**A**) $^{14}NH_4Cl$ and (**B**) $^{15}NH_4Cl$. (**C**) Resulting calibration curves for $^{14}NH_4Cl$ and $^{15}NH_4Cl$. (**D**) Signal obtained for the ^{1}H NMR quantification of the electrode deposit resulting from the electrolysis (10 C charge) of a $Mg(NTf_2)_2$ 0.5 M in DME electrolyte saturated with $^{15}N_2$, confirming that no $^{15}N_2$ was fixated.

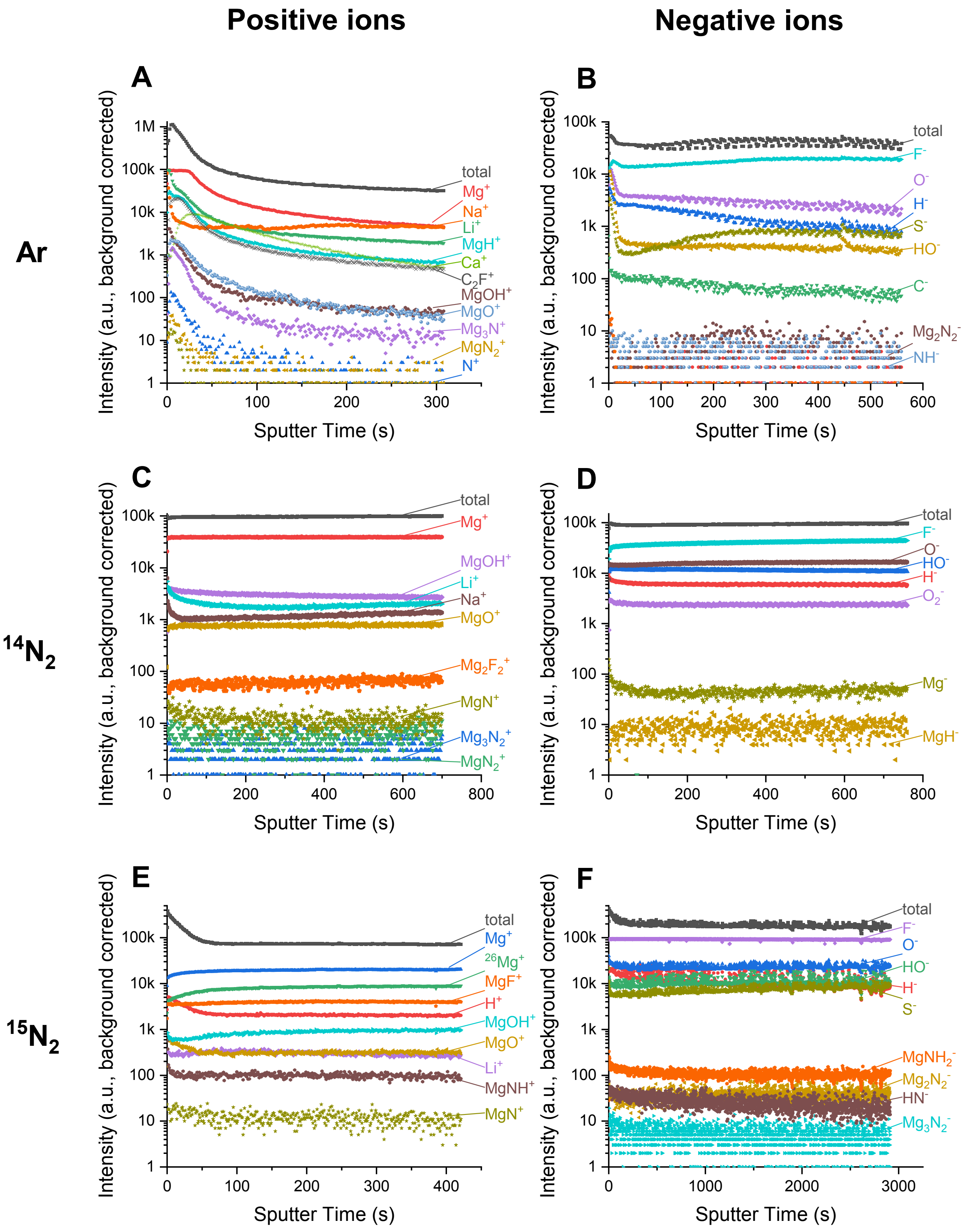

Positive ions
Negative ions
Ar
$^{14}N_2$
$^{15}N_2$
A
B
C
D
E
F
Intensity (a.u., background corrected)
Sputter Time (s)
total
Mg^+
Na^+
Li^+
MgH^+
Ca^+
C_2F^+
$MgOH^+$
MgO^+
Mg_3N^+
MgN_2^+
N^+
F^-
O^-
H^-
S^-
HO^-
C^-
$Mg_2N_2^-$
NH^-
$Mg_2F_2^+$
MgN^+
$Mg_3N_2^+$
O_2^-
Mg^-
MgH^-
$^{26}Mg^+$
MgF^+
H^+
$MgNH^+$
$MgNH_2^-$
HN^-
$Mg_3N_2^-$

Figure S 6. Time-of-Flight - Secondary Ion Mass Spectrometry (ToF-SIMS) depth profiling of the electrode deposits obtained after electrolysis of $Mg(NTf_2)_2$ 0.5M in DME.

(**A, C, E**) Positive ions and (**B, D, F**) negative ions detected on electrode deposits resulting from electrolysis in (**A, B**) Argon, (**C, D**) $^{14}N_2$ and (**E, F**) $^{15}N_2$. Results highlight three main points. First, the electrode surface contains Mg oxide and fluoride species, which are known to passivate Mg electrodes in batteries. Second, Mg nitride fragments can be observed; however, such nitrides are observed in all atmospheres, suggesting that they originate from the breakdown of NTf_2^- ions. Finally, the system is contaminated with traces of Li, Na and Ca, which is often the case in commercial alkali metal salts.

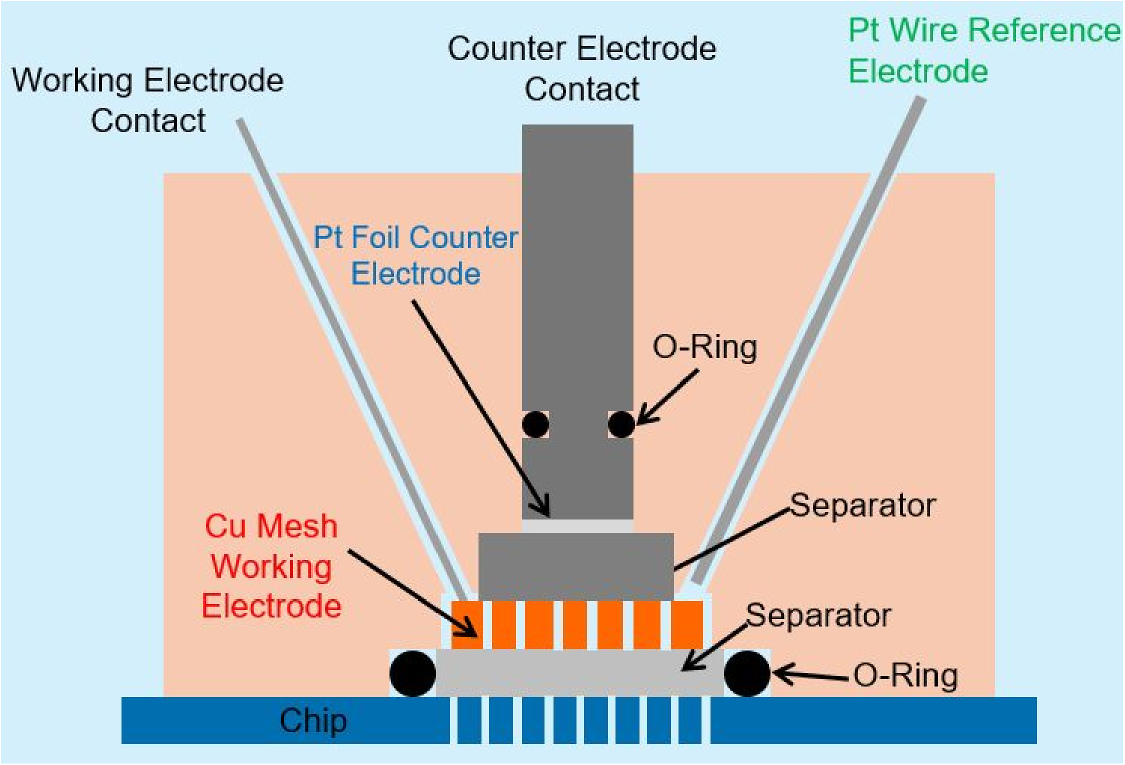

Figure S 7. Schematic of the Electrochemistry – Mass Spectrometry cell for in line gas evolution analysis.

The Spectro Inlets Electrochemistry-Mass Spectrometry system is housed inside an N5 Argon glovebox. The membrane and capillary are fabricated using semiconductor fabrication techniques and are contained within a thumb-sized silicon chip. The electrochemical cell sits on top of the silicon chip and has a disc-shaped working volume in which the electrodes and separators are housed. The working volume has a diameter of 9 mm and a thickness of 100 μm. The layers inside the cell are assembled in ascending order: Celgard® 2500 separator, EL-Cell

GmbH copper mesh, Whatman® glass microfiber filter GF/A grade, MaTecK Pt foil counter electrode, stainless steel plunger (counter electrode contact). The cell has 3 ports: one for a Pt wire (acting as the reference electrode), one for a copper wire (working electrode contact), one for electrolyte injection. More details are available in the methods section.

4. Tables

Table S 1. Summary of the theoretical data gathered from the literature or calculated.

Metal nitrides-hydrides structures and Gibbs free energies of their formation and N* binding to metals were calculated by DFT by us. Lewis acidity hardness/softness character was obtained by Klopman via quantum mechanical calculations[13]. Elements representative minerals global productions were collected from the US Geological Survey – Mineral Commodity Summaries 2018 to 2022[14]. SHE potentials were obtained from multiple references[15–18].

Element	M_xH_y	M_xN_y	Cleaved Nitride?	M_xH_y Formation Energy (eV/atom)	M_xN_y Formation Energy (eV/atom)	N* binding energy (eV)	Global Mining (kton/year)	Standard Reduction Potential (V *vs.* SHE)
**Ag**	AgH	AgN_3	No	0.267	0.729	3.683	24.00	0.80
**Al**	AlH_3	AlN	Yes	0.036	-1.182	-0.313	68000.00	-1.66
**Au**	-	AuN_2	No		0.469	3.506	3.00	1.52
**B**	B_9H_{11}	BN	Yes	-0.084	-1.143		2900.00	-1.79
**Ba**	BaH_2	BaN_2	No	-0.461	-0.360	-0.650	7300.00	-2.91
**Be**	BeH_2	Be_3N_2	Yes	-0.006	-0.886	1.553	0.26	-1.85

**Ca**	CaH_2	Ca_3N_2	Yes	-0.472	-0.612	-1.041	1500000.00	-2.87
**Cd**	CdH	CdN_6	No	0.511	0.583	2.549	24.00	-0.40
**Co**	Co_4H	CoN	Yes	0.039	0.306	0.851	170.00	-0.28
**Cs**	CsH	CsN_3	No	-0.113	-0.038	2.665		-3.03
**Cu**	CuH	Cu_3N	No	0.194	0.368	2.314	26000.00	0.34
**Fe**	FeH	FeN	Yes	0.101	0.136	-0.666	3300000.00	-0.44
**Ga**	GaH	GaN	Yes	0.504	-0.263		0.43	-0.53
**Hf**	HfH_2	HfN	Yes	-0.372	-1.479	-2.095	34.00	-1.72
**Hg**	-	HgN_3	No		0.682		0.30	0.85
**In**	InH	InN	Yes	0.500	0.232		0.92	-0.34
**Ir**	-	IrN_3	No		0.573	0.978	0.03	1.00
**K**	KH	KN_3	No	-0.159	-0.027	2.873	46000.00	-2.93
**Li**	LiH	Li_3N	Yes	-0.346	-0.252	-0.169	100.00	-3.04
**Mg**	MgH_2	Mg_3N_2	Yes	-0.124	-0.599	0.012	950.00	-2.37
**Mn**	$Mn_{29}H_2$	MnN	Yes	0.018	-0.072	2.201	20000.00	-1.19
**Mo**	MoH	MoN	Yes	0.544	-0.231	-1.115	16000.00	-0.15
**Na**	NaH	NaN_3	No	-0.146	0.013	1.969	290000.00	-2.71
**Nb**	NbH_2	NbN	Yes	-0.163	-0.987	-1.251	75.00	-1.10
**Ni**	NiH	Ni_6N_2	Yes	0.068	0.211	-0.098	2700.00	-0.25
**Os**	-	OsN_2	No		0.463	0.874	0.01	0.65
**Pd**	-	PdN_2	No		0.392	1.451	0.05	0.92
**Pt**	-	PtN_2	No		0.690	1.279	0.27	1.19
**Rb**	RbH	RbN_3	No	-0.112	-0.022	3.046		-2.98
**Re**	ReH_3	ReN	Yes	0.611	0.191	-0.210	0.06	0.30
**Rh**	-	RhN_2	No		0.597	0.895	0.02	0.76
**Ru**	-	RuN	Yes		0.404	0.725	0.09	0.46
**Sc**	ScH_2	ScN	No	-0.584	-1.729	-1.641	0.27	-2.08
**Sr**	SrH_2	SrN_2	No	-0.455	-0.279	-0.358	360.00	-2.90
**Ta**	TaH_2	TaN	Yes	-0.001	-0.973	-1.632	2.10	-0.60
**Ti**	TiH_2	TiN	Yes	-0.392	-1.511	-2.001	210.00	-1.63
**Tl**	TlH	TlN_3	No	0.455	0.420	2.909	2.10	-0.34
**V**	VH_2	VN	Yes	-0.109	-0.920	-1.526	110.00	-1.13
**W**	-	W_2N_3	Yes		-0.268	-1.020	79.00	-0.12
**Y**	YH_2	YN	Yes	-0.627	-1.554	-1.635	0.27	-2.37
**Zn**	ZnH_6	Zn_3N_2	Yes	0.148	0.247	1.843	13000.00	-0.76
**Zr**	ZrH_2	ZrN	Yes	-0.486	-1.574	-2.110	1200.00	-1.45

References

(1) Tort, R.; Westhead, O.; Spry, M.; Davies, B. J. V.; Ryan, M. P.; Titirici, M.-M.; Stephens,

I. E. L. Nonaqueous Li-Mediated Nitrogen Reduction: Taking Control of Potentials. *ACS Energy Lett.* **2023**, *8*, 1003–1009. https://doi.org/10.1021/acsenergylett.2c02697.

(2) McShane, E. J.; Benedek, P.; Niemann, V. A.; Blair, S. J.; Kamat, G. A.; Nielander, A. C.; Jaramillo, T. F.; Cargnello, M. A Versatile Li0.5FePO4 Reference Electrode for Nonaqueous Electrochemical Conversion Technologies. *ACS Energy Lett.* **2022**, *11*, 230–235. https://doi.org/10.1021/ACSENERGYLETT.2C02190.

(3) Nielander, A. C.; Blair, S. J.; McEnaney, J. M.; Schwalbe, J. A.; Adams, T.; Taheri, S.; Wang, L.; Yang, S.; Cargnello, M.; Jaramillo, T. F. Readily Constructed Glass Piston Pump for Gas Recirculation. *ACS Omega* **2020**, *5* (27), 16455–16459. https://doi.org/10.1021/acsomega.0c00742.

(4) Westhead, O.; Spry, M.; Bagger, A.; Shen, Z.; Yadegari, H.; Favero, S.; Tort, R.; Titirici, M.; Ryan, M. P.; Jervis, R.; Katayama, Y.; Aguadero, A.; Regoutz, A.; Grimaud, A.; Stephens, I. E. L. The Role of Ion Solvation in Lithium Mediated Nitrogen Reduction. *J. Mater. Chem. A* **2023**. https://doi.org/10.1039/D2TA07686A.

(5) Mei, H.; Laws, T. S.; Terlier, T.; Verduzco, R.; Stein, G. E. Characterization of Polymeric Surfaces and Interfaces Using Time-of-flight Secondary Ion Mass Spectrometry. *J. Polym.*

Sci. **2022**, *60* (7), 1174–1198. https://doi.org/10.1002/pol.20210282.

(6) Trimarco, D. B.; Scott, S. B.; Thilsted, A. H.; Pan, J. Y.; Pedersen, T.; Hansen, O.; Chorkendorff, I.; Vesborg, P. C. K. Enabling Real-Time Detection of Electrochemical Desorption Phenomena with Sub-Monolayer Sensitivity. *Electrochim. Acta* **2018**, *268*, 520–530. https://doi.org/10.1016/j.electacta.2018.02.060.

(7) Krempl, K.; Hochfilzer, D.; Cavalca, F.; Saccoccio, M.; Kibsgaard, J.; Vesborg, P. C. K.; Chorkendorff, I. Quantitative Operando Detection of Electro Synthesized Ammonia Using Mass Spectrometry. *ChemElectroChem* **2022**. https://doi.org/10.1002/celc.202101713.

(8) Jain, A.; Ong, S. P.; Hautier, G.; Chen, W.; Richards, W. D.; Dacek, S.; Cholia, S.; Gunter, D.; Skinner, D.; Ceder, G.; Persson, K. A. Commentary: The Materials Project: A Materials Genome Approach to Accelerating Materials Innovation. *APL Mater.* **2013**, *1* (1), 11002. https://doi.org/10.1063/1.4812323/119685/.

(9) Hammer, B.; Hansen, L. B.; Nørskov, J. K. Improved Adsorption Energetics within Density-Functional Theory Using Revised Perdew-Burke-Ernzerhof Functionals. *Phys. Rev. B* **1999**, *59* (11), 7413–7421. https://doi.org/10.1103/PhysRevB.59.7413.

(10) Hjorth Larsen, A.; Jørgen Mortensen, J.; Blomqvist, J.; Castelli, I. E.; Christensen, R.;

Dułak, M.; Friis, J.; Groves, M. N.; Hammer, B.; Hargus, C.; Hermes, E. D.; Jennings, P. C.; Bjerre Jensen, P.; Kermode, J.; Kitchin, J. R.; Leonhard Kolsbjerg, E.; Kubal, J.; Kaasbjerg, K.; Lysgaard, S.; Bergmann Maronsson, J.; Maxson, T.; Olsen, T.; Pastewka, L.; Peterson, A.; Rostgaard, C.; Schiøtz, J.; Schütt, O.; Strange, M.; Thygesen, K. S.; Vegge, T.; Vilhelmsen, L.; Walter, M.; Zeng, Z.; Jacobsen, K. W. The Atomic Simulation Environment—a Python Library for Working with Atoms. *J. Phys. Condens. Matter* **2017**, *29* (27), 273002. https://doi.org/10.1088/1361-648X/aa680e.

(11) Enkovaara, J.; Rostgaard, C.; Mortensen, J. J.; Chen, J.; Dułak, M.; Ferrighi, L.; Gavnholt, J.; Glinsvad, C.; Haikola, V.; Hansen, H. A.; Kristoffersen, H. H.; Kuisma, M.; Larsen, A. H.; Lehtovaara, L.; Ljungberg, M.; Lopez-Acevedo, O.; Moses, P. G.; Ojanen, J.; Olsen, T.; Petzold, V.; Romero, N. A.; Stausholm-Møller, J.; Strange, M.; Tritsaris, G. A.; Vanin, M.; Walter, M.; Hammer, B.; Häkkinen, H.; Madsen, G. K. H.; Nieminen, R. M.; Nørskov, J. K.; Puska, M.; Rantala, T. T.; Schiøtz, J.; Thygesen, K. S.; Jacobsen, K. W. Electronic Structure Calculations with GPAW: A Real-Space Implementation of the Projector Augmented-Wave Method. *J. Phys. Condens. Matter* **2010**, *22* (25), 253202. https://doi.org/10.1088/0953-8984/22/25/253202.

(12) Mortensen, J. J.; Hansen, L. B.; Jacobsen, K. W. Real-Space Grid Implementation of the Projector Augmented Wave Method. *Phys. Rev. B* **2005**, *71* (3), 035109. https://doi.org/10.1103/PhysRevB.71.035109.

(13) Klopman, G. Chemical Reactivity and the Concept of Charge- and Frontier-Controlled Reactions. *J. Am. Chem. Soc.* **1968**, *90* (2), 223–234. https://doi.org/10.1021/ja01004a002.

(14) Multiple Authors. *Mineral Commodity Summaries 2022*; 2022. https://doi.org/10.3133/MCS2022.

(15) Lide, D. R. *CRC Handbook of Chemistry and Physics*, 87th ed.; CRC Press/Taylor and Francis Group, Ed.; 2006, 8-23.

(16) Haynes, W. M. *CRC Handbook of Chemistry and Physics*, 92nd ed.; Press, C., Ed.; 2011, 5-80-9.

(17) Atkins, P. W. *Inorganic Chemistry*, 5th ed.; Freeman, W. H., Ed.; 2010, 153.

(18) Bard, A. J.; Parsons, R.; Jordan, J. *Standard Potentials in Aqueous Solutions*; CRC Press, Ed.; 1985, 39-762.
